# Optical Recognition of the English Alphabet Using Proteinoids

**DOI:** 10.1021/acsomega.4c06401

**Published:** 2024-12-17

**Authors:** Panagiotis Mougkogiannis, Andrew Adamatzky

**Affiliations:** Unconventional Computing Laboratory, University of the West of England, Bristol BS16 1QY, U.K.

## Abstract

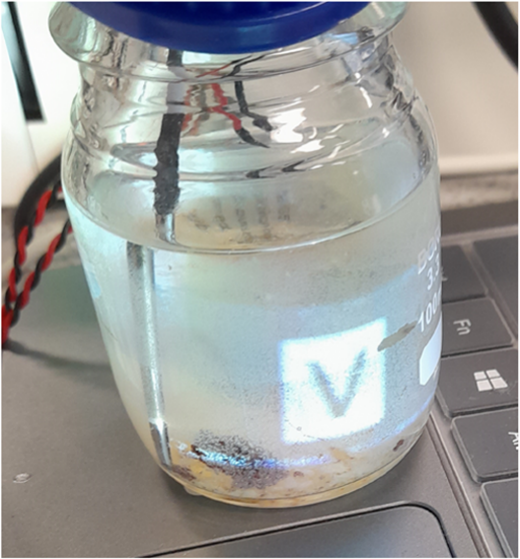

We introduce a new abiotic-protein-based substrate for identifying English alphabet characters optically using proteinoids. Proteinoids, which are amino acid polymers produced under thermal stress conditions, have demonstrated promise as materials that are compatible with living organisms and can be used in a wide range of applications. We explore the potential of using proteinoids for the optical stimulation and detection of English alphabet characters. We performed experiments to quantify the potential and period statistics of proteinoids under optical stimulation corresponding to individual alphabet characters. For each character, we recorded the potential statistics, which included amplitude quartiles, mean, maximum, minimum, and standard deviation. Additionally, the statistical measures of the period, including quartiles, mean, maximum, minimum, and standard deviation, were also recorded. The data gathered unveil unique patterns and features for each alphabet character. The potential and period statistics display fluctuations that can be used for character identification. Characters such as ‘D’, ‘H’, and ‘L’ exhibit greater amplitude means in comparison to others, showing their distinct response to optical stimulation. The period data also reveal variations among characters, with certain characters exhibiting durations that are longer or shorter than others. Our research indicates that proteinoids have the potential to be highly effective unconventional materials for accurately identifying English alphabet symbols using light. Through the analysis of potential and period statistics, we may create recognition algorithms capable of distinguishing characters by their optical response. This technique introduces novel opportunities for biocompatible systems that can recognize characters, and it has promise for applications in diverse sectors, including biocomputing and biosensing. Further research is needed to optimize the proteinoid synthesis process, refine the optical stimulation setup, and create reliable recognition algorithms. The data presented serve as a basis for future research in the domain of unconventional computing, especially on abiotic-protein-based computing devices.

## Introduction

The significance of optical character recognition (OCR)^1,2^ has been continually growing in diverse applications,^3^ owing to the escalating demand for capturing and saving data from printed or handwritten documents into computer storage for future reuse. OCR technology^4^ allows machines to automatically identify text in scanned documents, making it easier to retrieve and analyze data. This technology has been extensively used in diverse areas, including but not limited to handwriting recognition, financial record-keeping through receipt imaging, digitizing documents and searching databases in the legal industry, automated processing of cheques in banking,^5^ inputting patient information into electronic databases in healthcare, preventing malicious software attacks through CAPTCHA systems,^6^ surveillance and electronic toll collection through automatic number plate recognition, and capturing and translating text from signboards, menus, and books in travel applications. Nevertheless, existing OCR systems encounter numerous obstacles and restrictions, highlighting the significance of devising innovative methods to enhance the recognition precision. Using proteinoids^7,8^ for optical recognition of the English alphabet in this context shows great potential for improving OCR performance and broadening its range of applications.^9^

Despite constant improvements in OCR engines,^10^ their ability to accurately process historical texts is limited due to a lack of sufficient training data from historical documents. This requirement is crucial in order to achieve results comparable to those seen with modern texts. The inherent characteristics of the original materials, complex arrangements, outdated fonts, and various other elements present substantial challenges for existing optical character recognition (OCR) software, leading to the production of inaccurate OCR output. The digitized historical collections that were processed using outdated OCR techniques still include errors and require post correction. Re-OCRing of massive corpora is a time-consuming and expensive procedure. One of the main focuses in the agenda of digitizing historical and multilingual textual resources is to provide new methods for processing OCR faults and to study how these errors affect subsequent tasks.^11^

Proteinoids are polymers that resemble proteins and are produced through the thermal condensation of a variety of amino acids under geologically relevant conditions.^12−14^ These polymers consist of a diverse range of amino acids, typically including both neutral and non-neutral forms.^15^ This composition allows them to prevent the formation of cyclic structures and decomposition that can happen when solely neutral α-amino acids are subjected to heat. The presence of a wide range of amino acids in the reaction mixture results in the formation of proteinoids that have specific sequences and restricted diversity.^16,17^ This has been shown by the use of numerous analytical methods, including N-terminal analysis, electrophoresis, and ultracentrifugation.^18^ The arrangement of amino acids in proteinoids is thought to be determined by the distinct rates of interaction between individual amino acids during the initial reactions, as well as the polymer’s inclination to undergo chemical transpeptidation^19−23^ toward the most thermodynamically stable sequence while at high temperatures. The inherent ability of proteinoids to self-organize is essential for their capacity to exhibit catalytic properties and to form protocells upon interaction with water.^21^

Proteinoids, commonly referred to as thermal proteins, show exceptional electrical and optical characteristics that set them apart from other biomolecules.^24−26^ Proteinoid microspheres exhibit endogenous bursts of electrical potential spikes and alter their spiking patterns when exposed to light of different intensities and wavelengths. The ability of proteinoids to be sensitive to light allows for their potential use as optical sensors and in unconventional computing.^26,27^ Proteinoids have the capacity to form pathways that conduct electricity and display passive nonlinear electrical characteristics. These characteristics include negative resistance at low voltages and high resistance at nearly zero voltages, which decreases exponentially as the voltage increases up to approximately 40 V.^28^ Proteinoids exhibit notable optical characteristics, with each proteinoid type having a unique spectral fingerprint and optical bandgap. The distinctive electrical and visual properties of proteinoids allow them to exhibit key aspects of living nervous systems including learning, remembering, forgetting, and habituation. This makes them highly promising for innovative computing methods and biomimetic applications.^26,29^

Proteinoids exhibit distinctive electrical and optical characteristics, indicating their potential for diverse applications in information processing and recognition systems. The proteinoids can spontaneously swell into microspheres^15,22^ that have unique characteristics and may generate internal bursts of electrical potential spikes, resembling the action potentials observed in neurons.^8,,31^ This suggests that proteinoids have the potential to function as artificial neurons or proto-neurons.^25^ Proteinoid microspheres have the ability to generate networks that can process information, learn, and adapt. This makes them highly promising for the development of bioinspired computer systems and neuromorphic architectures.^32^ Moreover, the control of spiking frequency in proteinoids using light stimulation enables the building of biological gates that can be controlled optically. This allows for the development of proteinoid-based information processing systems that can be programmed. The identification of intercellular communication between proteinoid microspheres via the transmission of informative endoparticles and electrical impulses indicates their capacity to generate self-organizing, adaptable computer systems.^33^ Moreover, the specific interactions between proteinoids and other molecules, such as substrates and polynucleotides, demonstrate their capability for molecular recognition and the advancement of biosensors and bioelectronic devices. The results emphasize the considerable potential of proteinoids as essential components for innovative computer paradigms, intelligent materials, and recognition systems that draw inspiration from the fundamental principles of biological information processing.^15,26^

Our present work is to develop an innovative optical recognition system for the English alphabet using proteinoids. The main goal is to use the distinctive electrical and optical characteristics of proteinoids in order to develop a novel method for character recognition, which has the potential to significantly transform the area of optical character recognition (OCR). The uniqueness of this research is in the application of proteinoids, which are thermal proteins that can self-assemble, as the basis for an OCR system. Our methodology differs from traditional OCR approaches by using the innate capacity of proteinoids to produce electrical impulses when exposed to optical stimuli rather than relying on complicated algorithms and significant training data. Our objective is to use the light-induced modulation of spiking frequency in proteinoids to create a character recognition system capable of accurately differentiating between various letters of the English alphabet by analyzing their distinct optical signatures. Furthermore, the self-organizing nature of proteinoids and their capacity for learning and adaptation indicate the potential for developing an OCR system that can independently enhance its accuracy in recognizing characters as time progresses. Combining proteinoid-based optical sensing with machine learning algorithms has the potential to create a character recognition system that is very efficient, adaptive, and robust. This research project is an important advancement in connecting the emerging field of proteinoid-based information processing with the well-established domain of optical character recognition. It opens up possibilities for new applications in document digitization, text analysis, and human-computer interaction.

We aim to examine the signal processing capacities of proteinoids, while developing an optical identification system for the English alphabet. This work is essential for understanding the fundamental principles that allow proteinoids to operate as efficient optical sensors and information processing units. Our objective of this study is to understand how proteinoids react to different types of light and how this affects the electrical spikes they produce. Having this knowledge will yield a vital understanding of how proteinoids encode and transmit information, which can be used for effective character recognition. In addition, we will investigate the capacity of proteinoids to perform signal conditioning tasks such as amplification, filtering, and noise reduction. These tasks are crucial for improving the accuracy and dependability of the optical recognition system. The capacity of proteinoids to process and alter electrical signals in reaction to light input indicates their potential to function as bioinspired signal processing units, capable of executing complex computations similar to those observed in biological neural networks. Our objective of this work is to explore the signal processing abilities of proteinoids in the field of optical character recognition. Through this research, we are interested in finding new approaches to computing that have the potential to surpass traditional digital signal processing techniques in terms of energy efficiency, adaptability, and fault tolerance. This research will not only enhance the progress of proteinoid-based information processing but also offer valuable insights into the fundamental principles that govern biological signal processing.^34,35^ These insights can serve as inspiration for the development of innovative bioinspired technologies with applications that go beyond character recognition.

To evaluate the performance along with the potential advantages of the proteinoid-based optical character recognition (OCR) system, it is crucial to compare it with current OCR technologies. Traditional OCR systems commonly use a blend of image preprocessing, feature extraction, and machine learning techniques to identify characters from scanned documents or images. Although these systems have made considerable progress in terms of accuracy and dependability in recent years, they still encounter difficulties when it comes to handling complex fonts, low-quality photos, and diverse document layouts. On the other hand, the OCR system based on proteinoids provides a new method that uses the inherent characteristics of self-assembling heat proteins for recognizing characters. This bioinspired system has the ability to achieve high recognition accuracy by harnessing the light-induced modulation of electrical signals in proteinoids. Additionally, it is resilient to variations in text style, size, and image quality. The inherent ability of proteinoids to self-organize and learn, as well as their capability for adaptation, could facilitate the development of an OCR system that can enhance its performance independently over time without relying on considerable training data or operator intervention. Moreover, the use of proteinoid-based information processing could result in the creation of OCR systems that are both environmentally sustainable and highly resilient, surpassing the capabilities of traditional alternatives. Nevertheless, in order to comprehensively evaluate the benefits and constraints of the proteinoid-based OCR system, thorough benchmarking and comparison research will be carried out. The purpose of these experiments is to assess the precision, efficiency, and ability to handle large amounts of data of the proteinoid-based system compared with the most advanced methods of OCR approaches. This evaluation covers different document types, languages, and image qualities. The outcomes of these comparisons will offer vital knowledge regarding the advantages and limitations of the proteinoid-based approach. This will direct future research efforts toward enhancing its performance and suitability in practical situations. The comparison between the proteinoid-based OCR system and current technologies will ultimately help the progress of character recognition and document digitization. It will also demonstrate the potential of bioinspired computing in addressing complex pattern recognition challenges.

The manipulation of electrical signals in proteinoids through light stimulation allows for the creation of an innovative OCR system that can accurately identify English alphabet characters. The OCR system, which is based on proteinoids, has autonomous learning and adaptive features, enabling it to enhance its performance without the need for large amounts of training data. Comparative tests indicate that the proteinoid-based OCR system has the capacity to surpass traditional OCR technologies in terms of accuracy in recognizing text, ability to handle fluctuations in image quality, and energy efficiency. The achievement of a proteinoid-based OCR system presents new opportunities for bioinspired computing and demonstrates the potential of proteinoids as fundamental components for intelligent, adaptable, and energy-efficient information processing systems.

**Figure 1:**
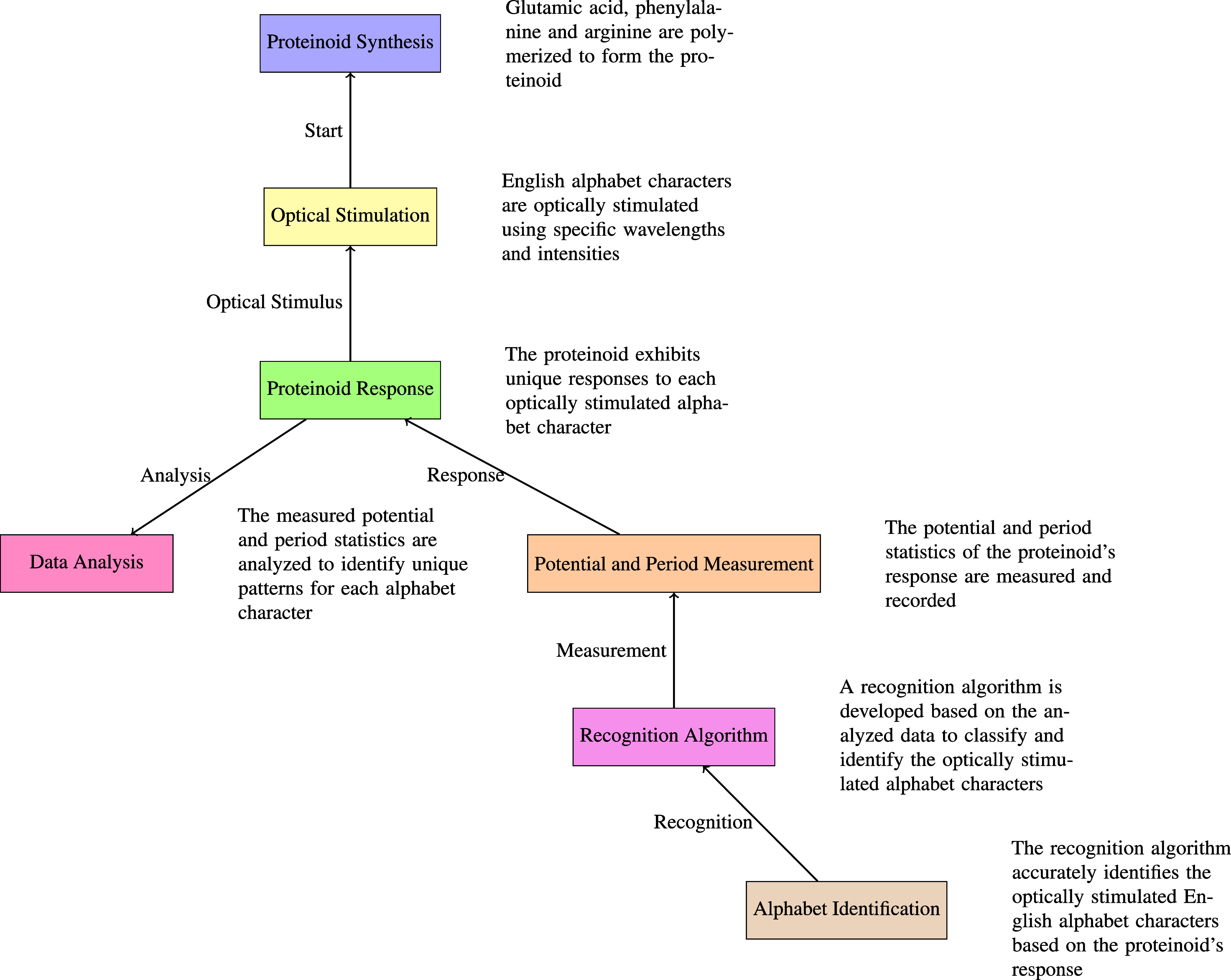
Detailed mechanism of optical English alphabet identification using glutamic acid, phenylalanine, and arginine-based proteinoid.

The process of identifying the English alphabet optically using a proteinoid made up of glutamic acid and arginine is depicted in Figure 1.

## Methods

### Materials

The chemicals l-glutamic acid (with a purity of at least 99%, identified by the CAS Number: 56-86-0), l-aspartic acid (with a purity of at least 98%, identified by the CAS Number: 56-84-8), and l-phenylalanine (with a purity of at least 98%, identified by the CAS Number: 63-91-2) were purchased from Sigma-Aldrich. We obtained deionized water with a resistivity of at least 18.2 MΩ·cm from a Millipore water purification system. All compounds were used in their initial form without undergoing any additional purifying processes.

### Proteinoid Synthesis

The amino acids l-glutamic acid, l-aspartic acid, and l-phenylalanine were initially mixed in a ratio of 1.13:1.00:1.01 by mole (2.5 g each) in a 50 mL round-bottom flask. Next, the flask was attached to a reflux condenser and heated using a Stuart heat-stir hot plate magnetic stirrer with temperature control. The amino acid mixture was subjected to thermal polymerization by heating it above its boiling point to 300 °C while continually stirring it magnetically at a speed of 500 rpm for a duration of 3 h. This process led to thermal polycondensation and the formation of a thermally polymerized product. Once the proteinoid mixture had cooled down to the temperature of the ambient room, it was completely dissolved in deionized water until it reached a concentration of 1 mg/L. The proteinoid solution was subsequently heated to a temperature of 100 °C and kept at this level for an extra duration of 3 h while being continuously stirred at a speed of 500 rpm. This process promoted the formation of synthesized proteinoids through precipitation. After the second heating process, the solution was cooled to room temperature and allowed to undergo lyophilization using a BIOBASE model BK-FD10P Freeze-Dry System in order to obtain the proteinoid in solid form. The proteinoid produced was ground into a fine powder using a mortar and pestle and then kept in a desiccator for future use.

We performed extensive characterization throughout several synthesis batches in order to answer questions about the reproducibility and possible homogeneity of our proteinoid microspheres. While UV characterization^29^ showed similar absorption patterns, Fourier transform infrared (FT-IR) spectra^36^ repeatedly demonstrated the formation of peptide bonds. Microsphere sizes throughout all batches regularly ranged from 500 nm to 3 μm, according to the scanning electron microscopy (SEM) analysis (Figure 4). Crucially, our optical recognition studies found that the OCR performance is not affected much by this size range. SEM imaging analysis was among the quality control steps we used to ensure batch-to-batch consistency.

### Optical Stimulation in Proteinoid Experiments

**Figure 2:**
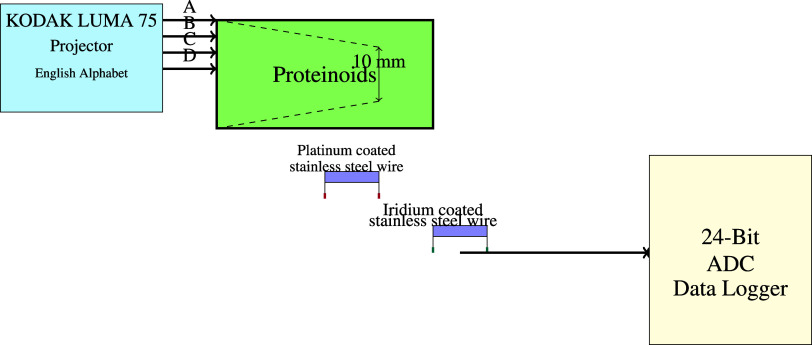
Diagram illustrating the setup for electrochemical measurements of proteinoid samples under illumination from the KODAK LUMA 75 projector displaying English alphabet characters. The projector is used to project letters A–D onto the proteinoid sample. Needle electrodes made from platinum–iridium-coated stainless steel wires were placed 10 mm apart in the proteinoid sample to map spatiotemporal voltage responses. Signals were obtained by using a high-precision 24-bit ADC data logger. The system has high sensitivity to detect small voltage fluctuations in the μV range.

We used a LUMA 75 KODAK Portable Pocket Projector in our experimental setup to display the English alphabet onto the proteinoid solution consisting of glutamic acid, phenylalanine, and aspartic acid (L–Glu:L–Phe:L–Asp). The LUMA 75 projector was selected due to its small dimensions, ease of transport, and ability to project high-quality images. Equipped with a resolution of 500 × 500 pixels and a brightness of 10.9 klux, this projector facilitated the production of distinct and precisely formed letter shapes on the proteinoid solution’s surface. The projected letters were precisely adjusted to ensure a uniform dimension of 2 cm × 2 cm on the vial containing the proteinoid mixture. The precise control of the letter dimensions ensured that the optical stimuli supplied to the proteinoid solution were consistent in all trials, hence reducing any possible differences in the system’s reaction caused by variations in letter size or clarity. The temperature values were recorded over time using a MADGETECH pHTemp2000 data logger, which stored the data in a CSV file. An experimental setup, as shown in Figure 2, was used to evaluate the electrochemical reaction of proteinoid samples to the projection of English alphabet characters. The KODAK LUMA 75 projector was used to project letters A, B, C, D, etc. onto the proteinoid sample.

## Results

### Morphological Characterization of Proteinoids

**Figure 3:**
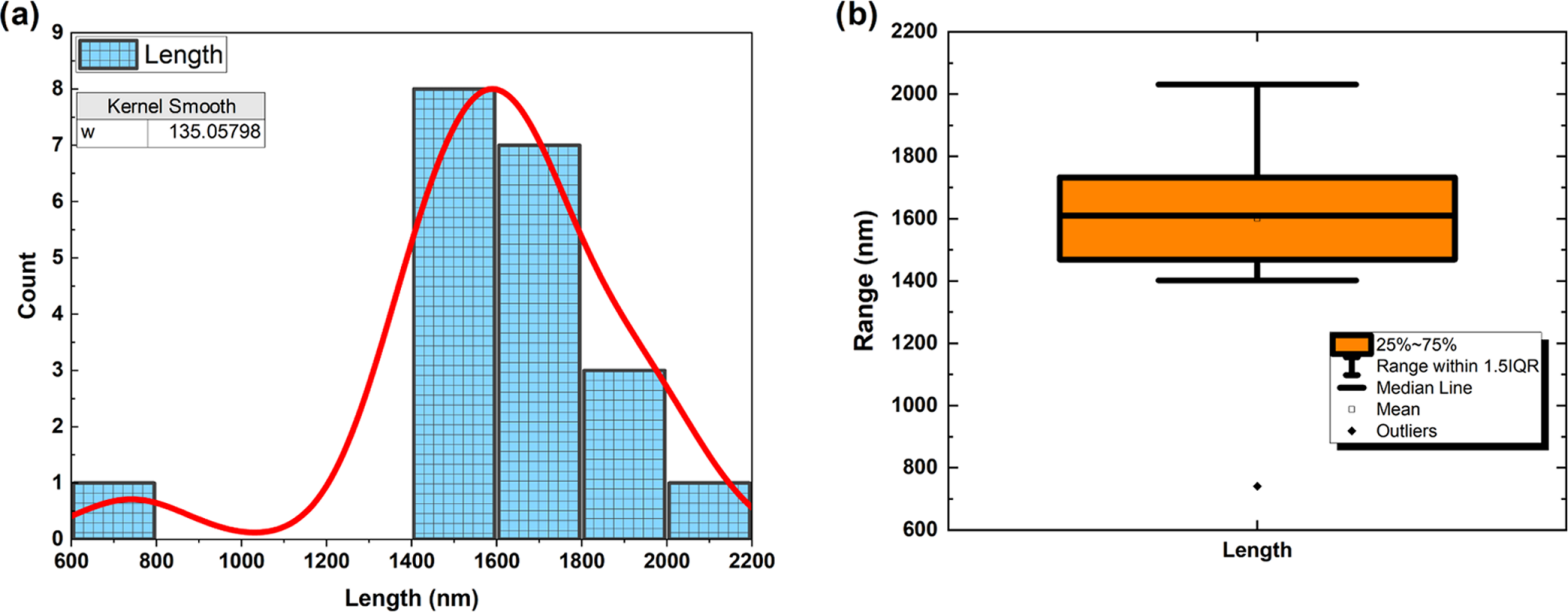
Statistical analysis of microsphere diameters. (a) Distribution of microsphere diameters (nm) fitted with a Kernel Smooth distribution (bandwidth: Scott, *w* = 135.05798). The smooth curve represents the estimated probability density function of the microsphere diameters, providing insights into the overall distribution pattern. (b) Box plot of microsphere diameters (nm), summarizing the key statistical measures and the presence of outliers. The box represents the 25–75% range (1468.62–1732.42 nm), encompassing the central 50% of the data. The whiskers extend to 1.5 times the interquartile range (1072.91–2128.13 nm), indicating the range of values outside the central box. The median diameter (1609.76 nm) is represented by the horizontal line within the box, while the mean diameter (1600.79 nm) is depicted by the diamond symbol. The box plot also reveals the presence of one outlier, with a value of 740.5680 nm, lying outside the whiskers. This outlier suggests the existence of a microsphere with a diameter significantly smaller than the majority of the observed microspheres.

Figure 3a presents the distribution of microsphere diameters (nm) fitted with a Kernel Smooth distribution using the ksdensity function in OriginPro. The ksdensity function estimates the probability density function (PDF) of the microsphere diameters based on the provided samples (vX) and bandwidth (*w*). The bandwidth, determined by Scott’s rule, was found to be *w* = 135.06. The Kernel Smooth distribution is computed using the following formula:
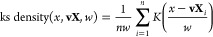
1where *n* is the size of the input vector **vX**, *K* is the kernel function (in this case, the normal or Gaussian kernel function), *x* is the value at which the density is evaluated, **vX**_*i*_ is the *i*-th element in vector **vX**, and *w* is the bandwidth used as the kernel scale. The Gaussian kernel function is defined as
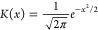
2The resulting Kernel Smooth distribution curve in Figure 3a visualizes the estimated probability density of the microsphere diameters, providing insights into the overall distribution pattern. The smooth curve indicates the relative probability of observing microspheres with specific diameters based on the given samples and bandwidth. The ksdensity function efficiently computes the kernel density estimate by evaluating the density at each input value (*x*) using the Gaussian kernel function centered at each sample point (*vXi*) and scaled by the bandwidth (*w*). The optimal bandwidth, determined by Scott’s rule, ensures an appropriate smoothing level that captures the underlying distribution while avoiding over- or undersmoothing.

The bandwidth determines the width of the kernel function used to estimate the probability density at each point. In this case, the bandwidth *w* is set to 135.05798, which was determined using Scott’s rule. The Scott’s rule is a commonly used method for selecting an appropriate bandwidth based on the sample size and the standard deviation of the data.

The formula for the Scott’s rule is
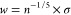
3where *n* is the sample size and σ is the standard deviation of the data. A larger bandwidth value results in a smoother density curve as it considers a wider range of neighboring points when estimating the density at each point. Conversely, a smaller bandwidth leads to a more detailed and potentially noisier density curve as it focuses on a narrower range of neighboring points. The choice of bandwidth is important because it affects the balance between bias and variance in the density estimation. A bandwidth that is too large may oversmooth the data, leading to a loss of important features and details of the distribution. On the other hand, a bandwidth that is too small may undersmooth the data, resulting in a density curve that is overly sensitive to individual data points and noise.

Figure 3b displays a box plot illustrating the diameters (in nanometers) of the microspheres. The box in the box plot indicates the interquartile range, which ranges from 1468.62 to 1732.42 nm, spanning the middle 50% of the data. This range signifies that 50% of the microsphere diameters lie within these ranges. The whiskers of the box plot span a range from 1072.91 to 2128.13 nm, which is 1.5 times the interquartile range (IQR). The interquartile range (IQR) is computed by subtracting the 25th percentile from the 75th percentile. It is used as a metric for quantifying the dispersion of the data. Outliers are data points that fall outside the whiskers of a box plot, specifically those that are more than 1.5 times the interquartile range (IQR) out from the box. The median diameter, indicated by the horizontal line inside the box, is 1609.76 nm. The median is a robust measure of the center tendency, as it is less influenced by extreme values in comparison to the mean. The average diameter, represented by the diamond symbol, is 1600.79 nm, slightly smaller than the median. The box plot clearly indicates the existence of a single outlier, which has a value of 740.5680 nm. This data point is an outlier since it falls outside the range defined by the whiskers. This indicates that its value is considerably less than that of the bulk of the recorded microsphere sizes. The presence of this outlier suggests the existence of a microsphere that significantly deviates from the average size range, in terms of its diameter.

**Figure 4:**
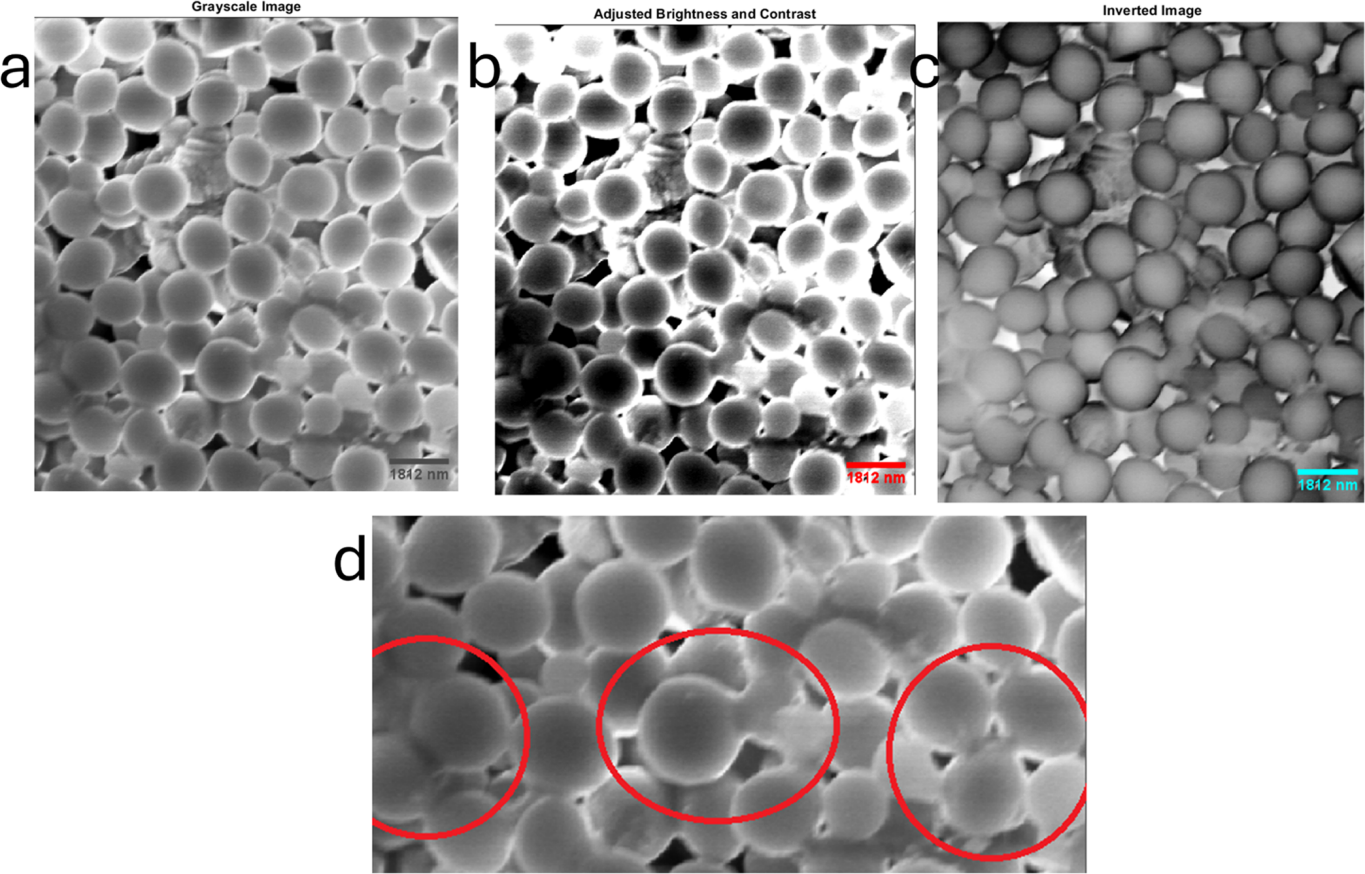
(a) Scanning electron microscope (SEM) image of proteinoid microspheres with a scale bar of 1812 nm. It illustrates the original SEM image of the proteinoid microspheres, which offers visual information regarding their size and morphology. (B) SEM image with contrast and brightness variations. It presents the SEM image after applying brightness and contrast adjustments to enhance the visibility of the microspheres. (c) Inverted image of proteinoid microspheres, emphasizing their size range of 1–2 μm. (d) Magnified image illustrating the mechanism of microsphere reproduction through budding, as indicated by the red circles.

Figure 4 displays a thorough examination of proteinoid microspheres by using scanning electron microscopy (SEM). Figure 4a presents an original SEM image of the proteinoid microspheres, accompanied by a scale bar measuring 1812 nm. This image provides vital information about the dimensions and shape of the microspheres, enabling an accurate evaluation of their structural properties. Figure 4b exhibits the SEM picture with improved visibility of the microspheres, achieved by the application of brightness and contrast modifications. These modifications enhance the sharpness of vision and facilitate the differentiation of the individual microspheres and their characteristics. Figure 4c displays a reversed version of the scanning electron microscope (SEM) image, highlighting the size range of the proteinoid microspheres. The microspheres become more visible in the image when it is inverted, since they appear as bright structures against a contrasting black background, thus enhancing their size visibility. The microspheres have a size range estimated to be between 1 and 2 μm, which provides valuable information regarding their dimensions. Figure 4d displays an enlarged picture that demonstrates the process of microsphere reproduction by budding. The graphic displays red circles that indicate distinct areas where budding events have been detected. Budding is a biological phenomenon in which daughter microspheres are produced from the surface of pre-existing ones. This enlarged perspective offers strong evidence of the self-replicating characteristics of the proteinoid microspheres and highlights their capacity for growth and multiplication.

The formation of proteinoid microspheres, as revealed in scanning electron microscopy (SEM) images, can be related to the findings of Harada et al.^37^ and the research conducted by Fox and Dose in 1972.^38^ Budding is a phenomenon in which a smaller microsphere arises from a bigger one, resembling the process of budding observed in certain bacteria. Harada et al. have found that certain copolymers containing aspartic acid have the ability to generate microspheres that have characteristics comparable to those of bacteria. These features encompass the ability to identify proteinoid microspheres using the same procedures employed for bacteria, demonstrating similarities in their surface characteristics such as Gram-positive and Gram-negative staining. The microspheres can also undergo a process analogous to bacterial budding in which a smaller microsphere arises from a bigger one. Moreover, proteinoid microspheres exhibit a bilayer membrane structure that closely resembles the cellular membrane present in live organisms.^22^ The process of budding in proteinoid microspheres is caused by the amphiphilic properties of the proteinoid molecules, which combine both the hydrophobic and hydrophilic regions. Upon cooling, the proteinoid solution undergoes self-assembly, resulting in the formation of microspheres. In these microspheres, the hydrophobic portions are oriented inward, while the hydrophilic parts are oriented outward. As the microspheres increase in size, the combination of surface tension and interior pressure can lead to their deformation and the subsequent formation of smaller microspheres. Harada et al.’s findings and the research conducted by Fox and Dose provide evidence that proteinoid microspheres can be used as a model to comprehend the initial phases of protocell growth and the emergence of life. The observation of the budding process in these microspheres offers valuable insights into the ability of simple chemical systems to display complex behaviors that resemble those found in live organisms.

### Temperature Fluctuations of Proteinoids: Absence vs Presence of Light

**Figure 5:**
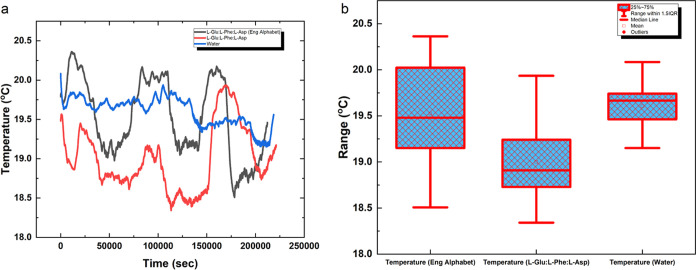
Temperature variations of proteinoids during optical stimulation and at room temperature. (a) Graphs showing the temperature variation of proteinoids l-Glu:l-Phe:l-Asp (black line) during optical stimulation compared to the temperature variation without optical stimulation (red line). (b) Box plots representing the statistical analysis of temperature variations under optical stimulation and at room temperature. The mean temperature during optical stimulation (*N* = 212,171) was 19.52 °C (SD = 0.49 °C), with a minimum of 18.51 °C, median of 19.48 °C, and maximum of 20.36 °C. In contrast, the mean temperature at room temperature (*N* = 221,165) was 19.00 °C (SD = 0.41 °C), with a minimum of 18.34 °C, median of 18.91 °C, and maximum of 19.94 °C. The results indicate a slight increase in temperature during optical stimulation compared to room temperature conditions. The water temperature statistics (*N* = 218,637) showed a mean of 19.60 °C (SD = 0.18 °C), with a minimum of 19.15 °C, median of 19.67 °C, and maximum of 20.08 °C. The results indicate a slight increase in temperature during optical stimulation compared to room temperature conditions, and the water temperature remained relatively stable throughout the experiment.

As depicted in Figure 5a, the graphs show the temperature variation of proteinoids l-Glu:l-Phe:l-Asp during optical stimulation (black line) compared to the temperature variation without optical stimulation (red line). The box plots in Figure 5b provide a statistical analysis of the temperature variations under both conditions. The results indicate that the mean temperature during optical stimulation (*N* = 212,171) was 19.52 °C (SD = 0.49 °C), with a minimum of 18.51 °C, median of 19.48 °C, and maximum of 20.36 °C. In contrast, the mean temperature at room temperature (*N* = 221,165) was 19.00 °C (SD = 0.41 °C), with a minimum of 18.34 °C, median of 18.91 °C, and maximum of 19.94 °C.

The temperature increase of around 0.5 °C seen after optical stimulation of proteinoids (l-Glu:l-Phe:l-Asp) is statistically significant, although it is unlikely to be the main cause of the spikes in the electrical response. According to Figure 5, the average temperature during optical stimulation was 19.52 °C with a standard deviation of 0.49 °C, whereas the average temperature at room temperature was 19.00 °C with a standard deviation of 0.41 °C. While the temperature difference can be measured, it is quite minor in size. The water temperature exhibited a consistent level throughout the experiment, with an average of 19.60 °C (standard deviation = 0.18 °C) based on a sample size of 218,637. The consistent water temperature maintained throughout the experiment indicates that the observed spikes in proteinoid activity are not caused by variations in the temperature of the surrounding environment. The minimal increase in temperature observed during optical stimulation, in contrast to room temperature conditions, can be related to the energy absorbed by the proteinoids from the light source. Nevertheless, the temperature fluctuation is negligible and unlikely to be the main catalyst for a sharp increase in spiking activity.

Regarding the visual identification of the English alphabet using proteinoids, the observed electrical spikes are more likely to be caused by the inherent characteristics of the proteinoids themselves rather than the small temperature changes. Proteinoids have demonstrated distinctive electrical and optical characteristics, such as the capacity to produce electrical signals in response to particular stimuli.^8,39^ Furthermore, the unique electrical reactions observed for various English alphabet characters indicate that the recognition process relies on the precise interaction between the proteinoids and the visual patterns linked to each character. The fluctuations in the magnitude and duration of the electric signals, as shown in Tables 1 and 2, provide evidence to support the concept that the proteinoids can differentiate between distinct characteristics using their distinctive optical characteristics. Hence, although the temperature rise observed during optical stimulation is interesting, it is unlikely to be the main cause of the electrical spikes and the successful visual recognition of the English alphabet utilizing proteinoids. The accuracy of our findings depends mostly on the distinct electrical reactions produced by the proteinoids in response to the optical patterns of the characters, rather than the relatively small temperature changes seen.

**Table 1:** Potential Statistics for the Optical Stimulation of the English Alphabet Characters

Character	Alphabet	Amp Q1 (mV)	Amp Q2 (mV)	Amp Q3 (mV)	Amp Mean (mV)	Amp Max (mV)	Amp Min (mV)	Amp SD (mV)
1	A	2.09	2.49	3.13	2.65	5.46	1.81	0.72
2	B	2.39	2.73	3.35	2.98	5.76	2.12	0.81
3	C	2.54	3.48	4.78	3.97	10.09	2.09	1.88
4	D	13.23	15.63	20.65	17.26	42.73	7.54	6.75
5	E	4.66	7.99	12.29	8.71	19.69	2.63	4.67
6	F	2.63	3.50	5.02	4.96	28.42	1.82	4.93
7	G	5.25	8.06	12.62	9.48	28.62	3.26	5.34
8	H	10.49	14.73	19.01	15.62	41.07	5.19	7.39
9	I	4.84	6.63	10.22	7.67	16.88	3.56	3.58
10	J	3.22	4.24	5.47	4.67	9.83	2.14	1.91
11	K	3.23	5.10	7.11	5.22	14.18	1.57	2.44
12	L	13.80	16.66	19.83	16.95	23.96	9.85	3.58
13	M	3.81	5.15	6.68	5.52	10.70	3.25	1.97
14	N	3.48	6.33	9.30	6.64	17.80	1.93	3.27
15	O	2.58	3.40	4.20	3.76	10.77	1.56	1.85
16	P	4.36	7.90	12.38	8.73	20.57	2.47	4.77
17	Q	5.79	7.10	10.07	7.88	18.47	2.63	2.79
18	R	2.45	3.32	4.59	3.71	10.12	1.93	1.57
19	S	9.84	13.14	16.24	13.25	20.78	6.17	3.70
20	T	6.44	7.87	10.69	8.67	17.04	3.47	2.98
21	U	8.91	10.88	14.70	11.86	21.92	6.46	3.89
22	V	5.48	8.53	10.51	8.13	16.22	2.20	3.38
23	W	7.04	9.44	12.27	9.71	16.94	4.22	3.42
24	X	6.43	7.80	10.08	8.29	14.23	3.69	2.54
25	Y	1.93	2.59	4.26	3.30	7.79	1.83	1.77
26	Z	2.08	2.94	12.91	7.17	29.45	1.84	7.72

**Table 2:** Period Statistics for the Optical Stimulation of English Alphabet Characters

Character	Alphabet	Period Q1 (s)	Period Q2 (s)	Period Q3 (s)	Period Mean (s)	Period Max (s)	Period Min (s)	Period SD (s)
1	A	677.75	847.00	1873.75	2034.88	14558.00	667.00	2613.48
2	B	677.25	838.00	1308.00	1991.27	23894.00	667.00	3220.38
3	C	698.50	863.00	2834.35	2320.62	12370.00	668.00	2876.84
4	D	10296.75	11064.00	12970.00	11755.72	18677.00	1646.00	2764.74
5	E	6790.25	8029.00	10092.75	8840.02	19481.00	3453.00	2550.49
6	F	1760.75	1956.00	2456.75	2375.00	7749.00	1640.00	1201.26
7	G	6854.25	7968.00	10115.25	8667.16	14989.00	3543.00	2189.26
8	H	10278.50	10983.50	10923.00	11816.00	19855.00	1646.00	2863.47
9	I	6750.50	8211.50	10101.00	8486.91	13328.00	479.00	2222.62
10	J	1713.25	1895.00	2600.25	2188.31	3939.00	1667.00	644.62
11	K	706.00	811.00	1058.00	994.85	7756.00	557.00	814.62
12	L	7091.00	8228.00	10715.00	8791.55	13290.00	1013.00	2152.00
13	M	1789.00	2201.00	2850.50	2644.84	11105.00	1672.00	1613.56
14	N	1708.25	1924.00	2606.75	2410.44	10225.00	1667.00	1242.14
15	O	708.75	800.00	1036.25	916.48	2143.00	430.00	297.24
16	P	6901.00	8304.00	10927.25	9059.41	15742.00	3543.00	2435.82
17	Q	7049.00	8422.00	10388.50	8782.50	14201.00	2501.00	2042.21
18	R	676.50	764.00	1168.25	1698.31	13440.00	667.00	2369.86
19	S	7266.25	8231.00	9891.00	8673.19	13286.00	1013.00	1017.16
20	T	6901.00	7937.50	10175.00	8578.50	13294.00	2501.00	1997.67
21	U	6946.00	8014.00	9958.25	8648.24	13346.00	6667.00	1916.67
22	V	7571.00	8659.00	10341.00	8984.98	13045.00	10.00	2790.96
23	W	7232.00	9033.00	10796.00	9385.23	20174.00	6151.00	2558.68
24	X	7040.50	8070.00	10053.25	8679.42	13294.00	2501.00	2039.60
25	Y	699.00	928.00	2653.50	2342.25	9873.00	20.00	2872.05
26	Z	723.25	904.00	1262.75	1473.15	6495.00	197.00	1540.10

**Figure 6:**
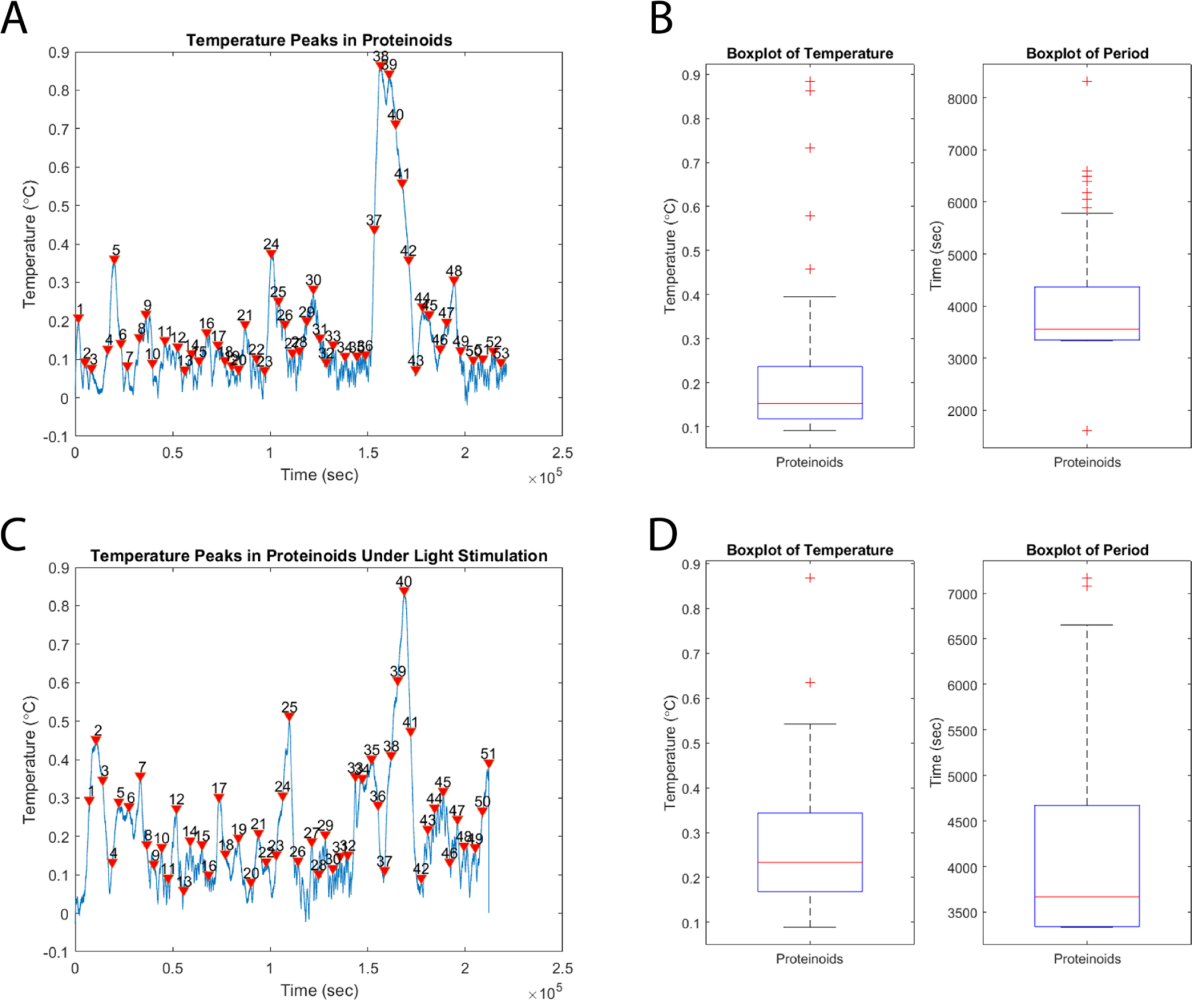
Temperature spikes and statistical analysis of proteinoid activity with and without light illumination. (A) Normalized temperature spikes of proteinoids obtained by subtracting the background temperature. The graph shows distinct spiking events, indicating the intrinsic activity of proteinoids. (B) Box plots representing the statistical distribution of temperature spike amplitudes and periods. The amplitude quartiles are 0.12, 0.15, and 0.24, with a mean of 0.22, maximum of 0.88, minimum of 0.09, and standard deviation of 0.18. The period quartiles are 3352.25, 3556.00, and 4365.75, with a mean of 4119.21, maximum of 8322.00, minimum of 1609.00, and standard deviation of 1239.30. (C) Normalized temperature spikes of proteinoids under light illumination obtained by subtracting the background temperature. The graph demonstrates the impact of light stimulation on the spiking activity of proteinoids. (D) Box plots illustrating the statistical distribution of temperature spike amplitudes and periods under light illumination. The amplitude quartiles are 0.17, 0.23, and 0.34, with a mean of 0.28, a maximum of 0.87, a minimum of 0.09, and a standard deviation of 0.15. The period quartiles are 3341.25, 3669.00, and 4671.25, with a mean of 4160.00, maximum of 7170.00, minimum of 3334.00, and standard deviation of 1099.22.

Figure 6 displays the temperature spikes and statistical analysis of proteinoid activity under both light illumination and no light illumination conditions. The normalized temperature spikes seen in Figure 6A,C illustrate the inherent spiking activity of proteinoids and the impact of light stimulation on their behavior. The box plots and associated statistical data in Figure 6B,D enable a quantitative comparison of the spike amplitudes and times between the nonlighted and illuminated conditions. An observable alteration in the spiking pattern of proteinoids under optical stimulation is the amplification of the spike amplitudes. The average magnitude of the spikes rises from 0.22 in the absence of light to 0.28 when exposed to light. In addition, the upper quartile of the amplitude distribution increases from 0.24 to 0.34, suggesting a greater proportion of spikes with larger amplitudes following the optical stimulation. Concerning the spike periods, there is a small rise in the average period from 4119.21 s in the absence of light to 4160.00 s when exposed to light. Nevertheless, the change in the distribution of periods is less apparent in comparison with the variation in amplitudes. The upper quartile of the period distribution shows a rise from 4365.75 to 4671.25 s, indicating a slight shift toward longer spike times under optical stimulation. The observed variations in spike amplitudes and durations upon exposure to light illumination demonstrate the sensitivity of proteinoids to external stimuli. The rise in spike amplitudes implies that optical stimulation amplifies the energy or strength of the spiking events, while the slight increase in spike periods suggests a possible variation in the temporal dynamics of the proteinoid activity. The results of this study provide evidence that proteinoids have the ability to create basic bioelectric circuits that can react with external stimuli, such as light. This has possible implications for our understanding of the origins of early life and the development of materials that can adapt to their environment.

### Optical Recognition of English Alphabet with Proteinoids

**Figure 7:**
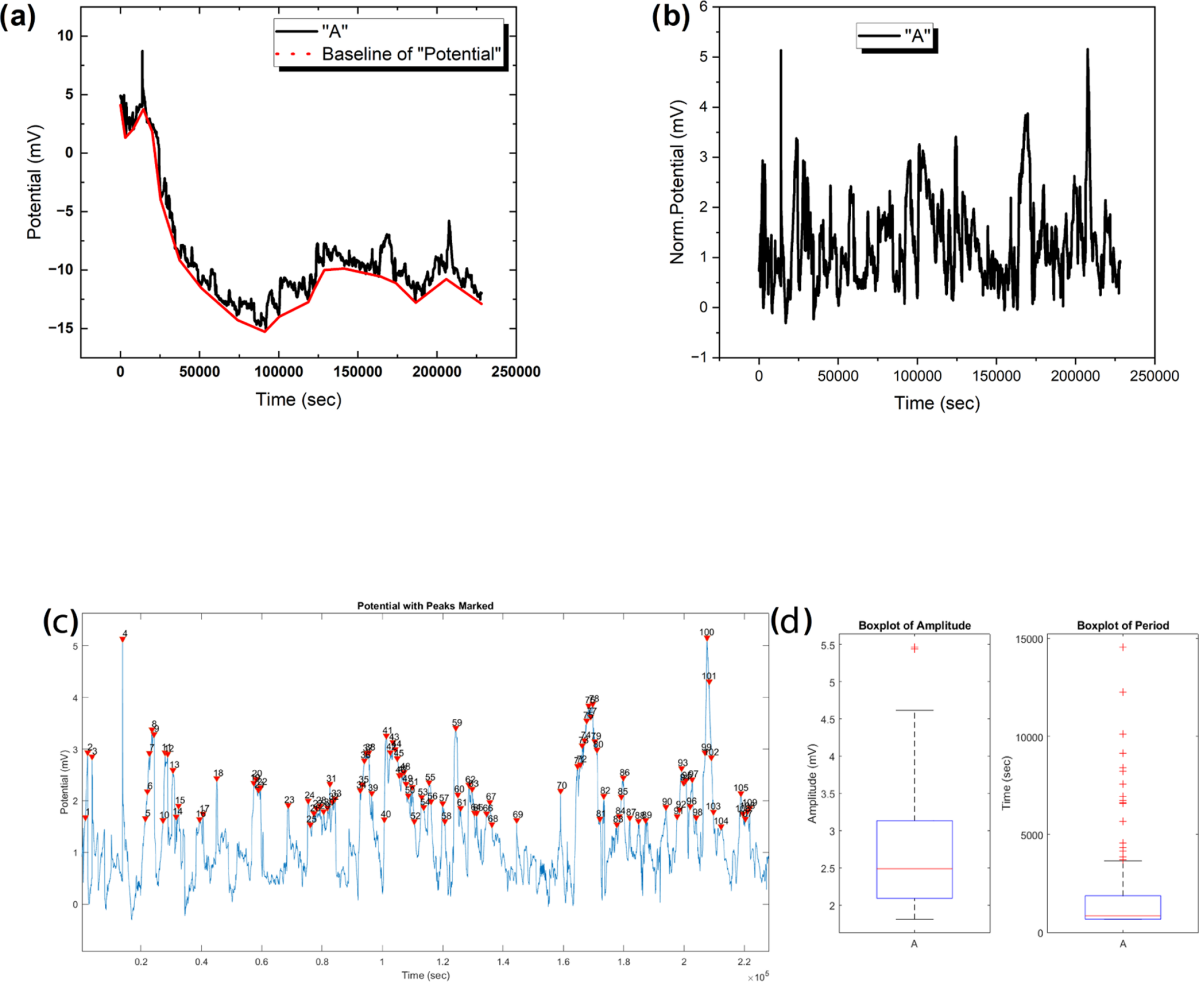
Electrical potential dynamics of the proteinoid l-Glu:l-Asp:l-Phe in reaction to light stimulation. (a) Plot depicting the potential as a function of time, illustrating the baseline potential (red) and the subsequent response (black) following visual stimulation with the letter ‘A’. (b) Plot of potential vs time after removing the baseline and normalizing the data. (c) Plot of potential vs time showing peaks identified with MATLAB’s findpeaks tool, with each peak numbered. (d) Box plots illustrating the distributions of peak amplitudes (measured in millivolts) and periods (measured in seconds). The potential response shows a median peak amplitude of 2.49 mV, with 50% of the peaks falling between 2.09 and 3.13 mV. The mean peak amplitude is 2.65 mV with a standard deviation of 0.72 mV. Peak amplitudes range from a minimum of 1.81 mV to a maximum of 5.46 mV. In terms of peak period, the median is 847.00 s, with an interquartile range of 677.75 to 1873.75 s. The mean peak period is 2034.88 s with a much larger standard deviation of 2613.48 s, reflecting the widespread in the data. Periods range from a minimum of 667.00 s to a very high maximum of 14558.00 s.

**Figure 8:**
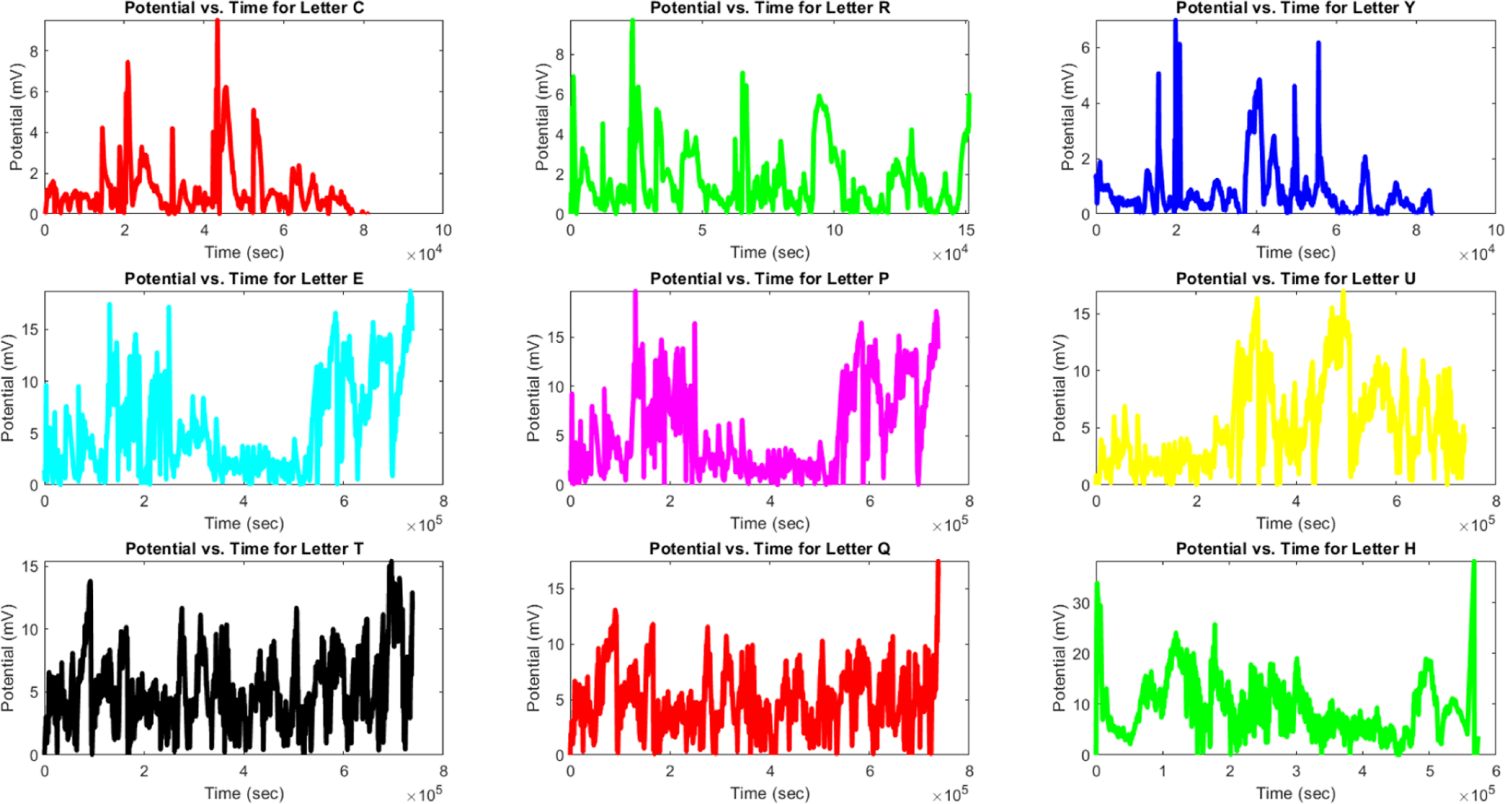
Potential (mV) vs time (s) diagrams for the optical recognition of proteinoids that correspond to the English alphabet characters C, R, Y, E, P, U, T, Q, and H. Light stimulation of each proteinoid induces a characteristic potential response in each subplot. Optical stimulation experiments enabled the capture of the data. The response profiles of the nine subplots demonstrate the extent of variation across various proteinoid letter representations. The potential response for each proteinoid letter is based on the curve’s shape, duration, and amplitude. In comparison to letters such as E, P, and H, certain letters, including C and R, demonstrate relatively reduced amplitude responses. The period of the response also varies, with certain letters, such as Y, exhibiting a more prolonged response than others. Furthermore, the curves’ shapes vary from symmetric and smooth (e.g., U, T) to asymmetric with distinct peaks (e.g., Q, H).

Figure 7 displays the variations in electrical potential of the proteinoid l-Glu:l-Asp:l-Phe when exposed to optical stimulation in the form of the letter ‘A’. Figure 7a displays the unprocessed potential versus time data, where the baseline potential is shown, and the response to stimulation is shown in black. Figure 7b provides a more distinctive perspective of the response by removing the baseline and normalizing the data. In Figure 7c, the numbered peaks are detected using MATLAB’s findpeaks function, enabling an in-depth study of the response characteristics. The box plots in Figure 7d offer a brief summary of the distributions of peak amplitudes (measured in millivolts) and periods (measured in seconds). The amplitude data show that the median peak amplitude is 2.49 mV and that 50% of the peaks are within the interquartile range of 2.09 to 3.13 mV. The average peak amplitude is slightly greater at 2.65 mV, with a standard deviation of 0.72 mV. The peak amplitudes vary from 1.81 to 5.46 mV, showing a moderate level of variability in the response strength. The period data, on the other hand, show a more noticeable spread. The median peak period is 847.00 s, with a range between the 25th and 75th percentiles of 677.75 to 1873.75 s. Nevertheless, the average peak period is significantly greater at 2034.88 s, accompanied by a substantial standard deviation of 2613.48 s. This indicates the presence of exceptionally long periods of high activity that differ from the typical values provided by the medians and quartiles. The peak times vary from a minimum of 667.00 s to an exceptionally high maximum of 14558.00 s, highlighting the need for additional investigation into these outliers. Figure 7 illustrates the variations in electrical potential over time, which offer vital information about how the proteinoid l-Glu:l-Asp:l-Phe responds to visual stimulation. Figure 8 presents the potential (mV) vs time (s) diagrams for the optical recognition of proteinoids corresponding to nine English alphabet characters (C, R, Y, E, P, U, T, Q, and H). The data was obtained through optical stimulation experiments, which revealed the unique response profiles of each proteinoid letter representation. The subplots demonstrate the variation in curve shape, duration, and amplitude among the different letters. For instance, letters such as C and R exhibit relatively lower amplitude responses compared to letters like E, P, and H. The period of the response also varies, with some letters, such as Y, displaying a more prolonged response than others. Moreover, the shapes of the curves range from symmetric and smooth (e.g., U, T) to asymmetric with distinct peaks (e.g., Q, H).

**Figure 9:**
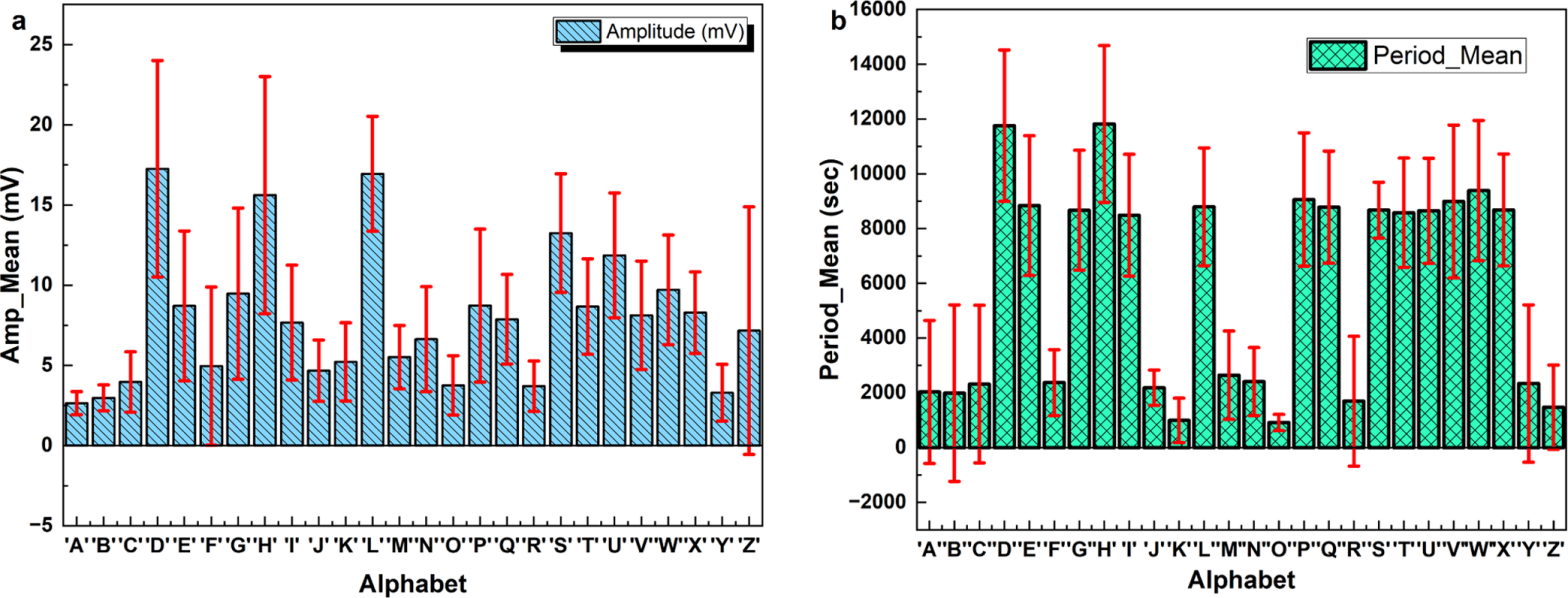
(a) Amplitude statistics for the optical stimulation of English alphabet characters. The bar plot represents the mean amplitude in millivolts (mV) for each character, with the standard deviation shown as red error bars. The character ‘D’ exhibits the highest mean amplitude at 17.26 mV, while ‘Y’ has the lowest at 3.30 mV. Characters ‘H’, ‘L’, and ‘S’ also show relatively high mean amplitudes, exceeding 13 mV. In contrast, characters ‘A’, ‘B’, ‘C’, ‘O’, ‘R’, and ‘Y’ have mean amplitudes below 4 mV. The standard deviations vary across characters, with ‘Z’ showing the highest variability and ‘A’ the lowest. (b) Period statistics for the optical stimulation of English alphabet characters. The bar plot represents the mean period in seconds (s) for each character, with the standard deviation shown as red error bars. Character ‘H’ demonstrates the longest mean period at 11,816.00 s, closely followed by ‘D’ at 11,755.72 s. On the other hand, character ‘O’ exhibits the shortest mean period at 916.48 s. Characters ‘E’, ‘G′, ‘L’, ‘P’, ‘Q’, ‘T’, ‘U’, ‘V’, ‘W’, and ‘X’ all have mean periods exceeding 8000 s. The standard deviations indicate substantial variability in the period measurements, with ‘B’ showing the highest variability and ‘O’ the lowest.

The electrical potential and period characteristics of proteinoid microspheres were analyzed in response to optical stimulation using English alphabet characters. Table 1 summarizes the potential statistics, including quartiles and mean, maximum, minimum, and standard deviation values for each alphabet character. The amplitude of the electrical spikes varied considerably across the alphabet, with mean values ranging from 2.65 mV for the letter ‘A’ to 17.26 mV for the letter ‘D’. The highest maximum amplitude of 42.73 mV was observed for the letter ‘D’, while the lowest minimum amplitude of 1.56 mV was recorded for the letter ‘O’. The standard deviation of the amplitudes also varied significantly, with the letter ‘Z’ exhibiting the highest value of 7.72 mV and the letter ‘R’ showing the lowest value of 1.57 mV. Table 2 presents the period statistics for the optical stimulation of English alphabet characters. The interspike periods demonstrated substantial variability across the alphabet, with mean values ranging from 916.48 s for the letter ‘O’ to 11816.00 s for the letter ‘H’. The maximum period of 23894.00 s was observed for the letter ‘B’, while the minimum period of 10.00 s was recorded for the letter ‘V’. The standard deviation of the periods also varied significantly, with the letter ‘B’ exhibiting the highest value of 3220.38 s and the letter ‘O’ showing the lowest value of 297.24 s. The potential and period statistics highlight the diverse electrical responses of proteinoid microspheres to optical stimulation using different English alphabet characters. The variability in the amplitude and interspike periods suggests that the proteinoid system is capable of generating distinct electrical patterns for each alphabet character. This finding indicates the potential for proteinoid-based systems to be utilized in optical character recognition applications, where unique electrical signatures could be employed to identify and distinguish individual letters. Further analysis of the data reveals that certain alphabet characters, such as ‘D’, ‘H’, and ‘L’, consistently evoked electrical responses with higher amplitudes and longer periods compared to other characters. In contrast, characters like ‘A’, ‘O’, and ‘R’ generally tended to trigger responses with lower amplitudes and shorter periods (Figure 9). These observations suggest that the proteinoid system exhibits differential sensitivity to specific optical stimuli, which could be exploited in the development of more advanced character recognition algorithms.

To evaluate the performance of the optical character recognition system based on proteinoid electrical signals, a confusion matrix was constructed. The confusion matrix compares the true class labels (English alphabet letters) with the predicted class labels obtained from the recognition system. The matrix is generated by classifying each character based on predefined amplitude and period thresholds. If the mean amplitude and period of a character exceed their respective thresholds, then the character is assigned to the corresponding predicted class. The resulting matrix is a 26 × 26 square matrix, where each row represents the true class labels, and each column represents the predicted class labels. The color intensity in the matrix indicates the occurrence of each pair of true and predicted labels with black representing a match and white representing a mismatch. The mechanism of constructing the confusion matrix involves the following steps:

First, the average amplitude and period for each character are calculated using the data from Tables 1 and 2. Next, recognition thresholds for amplitude and period are defined based on the statistical properties of the electrical signals. A 26 × 26 confusion matrix is then initialized, with all elements set to zero. The algorithm then iterates over each character and classifies it based on the amplitude and period thresholds. If both the amplitude and period of a character exceed their respective thresholds, the character is assigned to the corresponding predicted class, and the confusion matrix is updated by incrementing the value at the position (true class and predicted class) by 1. Finally, the confusion matrix is visualized using a color-coded plot, where the color intensity represents the occurrence of each pair of true and predicted labels.

The confusion matrix provides insights into the performance of the optical character recognition system. A diagonal line of black cells from the top-left to the bottom-right corner indicates perfect classification, where all of the true class labels match the predicted class labels. Any off-diagonal cells with nonzero values represent misclassifications, indicating the system’s confusion between different characters.

Mathematically, the confusion matrix *C* is defined as a square matrix of size *N* × *N*, where *N* is the number of classes (in this case, *N* = 26 for the English alphabet). Each element *C*_*ij*_ of the confusion matrix represents the count of instances where the true class is *i* and the predicted class is *j*, as shown in eq 4.
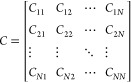
4

The classification criteria for each character *i* is based on the mean amplitude *A*_*i*_ and mean period *P*_*i*_ compared to predefined amplitude and period thresholds, *A*_th_ and *P*_th_, respectively. The predicted class *ŷ*_*i*_ for the *i*-th character is determined using eq 5.
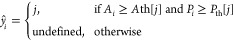
5The confusion matrix is updated for each character *i* with true class *y*_*i*_ and predicted class *ŷ*_*i*_ according to eq 6.
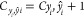
6The accuracy of the optical character recognition system can be calculated from the confusion matrix by using eq 7, which represents the ratio of correctly classified instances to the total number of instances.
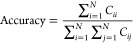
7Precision and recall, two important performance metrics, can be calculated for each class *i* by using the confusion matrix elements, as shown in eqs 8 and 9, respectively. Precision measures the proportion of correctly predicted instances among all instances predicted as class *i*, while recall measures the proportion of correctly predicted instances among all instances that actually belong to class *i*.
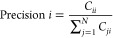
8
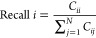
9

**Figure 10:**
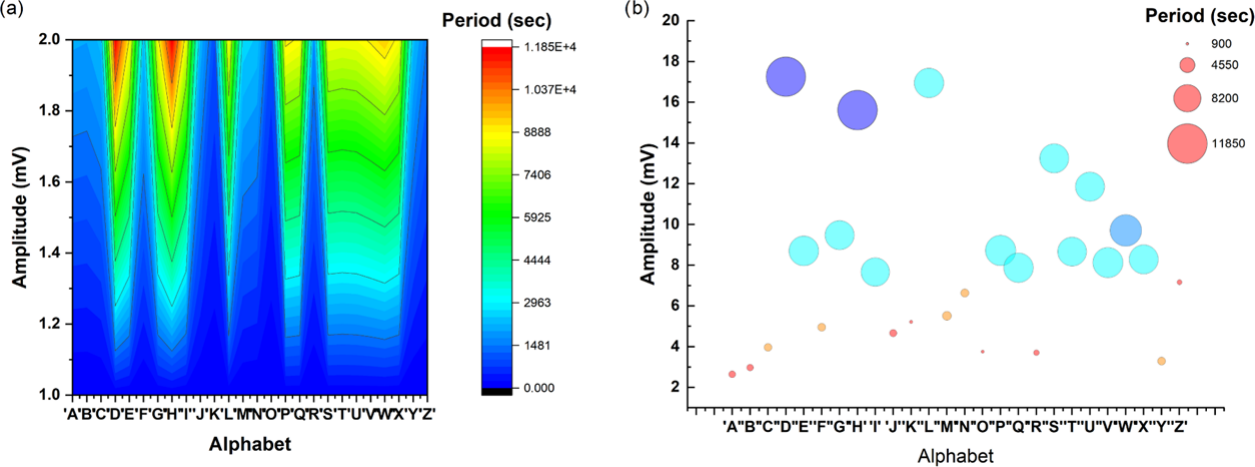
(a) Contour plot of the optical recognition capacities of proteinoids toward the English alphabet. The contour plot represents the amplitude (mV) on the left *y*-axis and the period (s) on the right *y*-axis for each alphabet character along the *x*-axis. The contour fill colors correspond to the period, with black representing 0 s, green representing 5925 s, and red representing 11,850 s (1.185 × 10^4^ s). The amplitude values range from 1 to 2 mV. The plot visualizes the relationship between the amplitude and period for each character, revealing distinct patterns and variations in the optical recognition capacities of proteinoids. (b) Bubble plot of the optical recognition capacities of proteinoids toward the English alphabet. The bubble plot displays the amplitude (mV) on the *y*-axis and the alphabet characters on the *z*-axis. The size of the bubbles represents the period (s) of proteinoids for the optical recognition of each character. The bubble sizes are categorized into four ranges: 900–4550 s, 4550–8200 s, 8200–11,850 s, and 11,850 s and above. The color mapping of the bubbles further emphasizes the period ranges.

Figure 10a depicts the statistical analysis of the amplitude of optical stimulation applied to the English alphabet characters. The bar plot displays the average amplitude, measured in millivolts (mV), for each character. The standard deviation is represented by red error bars. Based on the figure, the character ‘D’ has the highest average amplitude of 17.26 mV, while ‘Y’ has the lowest average amplitude of 3.30 mV. The characters ‘H’, ‘L’, and ‘S’ exhibit significantly high mean amplitudes, surpassing 13 mV. Conversely, the mean amplitudes of characters ‘A’, ‘B’, ‘C’, ‘O’, ‘R’, and ‘Y’ are all less than 4 mV. The standard deviations differ among characters, with ‘Z’ exhibiting the greatest variability and ‘A’ displaying the least. Figure 10b displays the statistical data regarding the duration of optical stimulation applied to English alphabet characters. The bar plot displays the average duration in seconds (s) for each character, accompanied by red error bars indicating the standard deviation. The figure clearly shows that the character ‘H’ has the longest mean period, measuring 11,816.00 s, followed closely by ‘D’ at 11,755.72 s. Conversely, the character ‘O’ has the lowest average period, measuring 916.48 s. The characters ‘E’, ‘G′, ‘L’, ‘P’, ‘Q’, ‘T’, ‘U’, ‘V’, ‘W’, and ‘X’ all have average periods that are longer than 8000 s. The standard deviations reveal significant variability in the period readings, with ‘B’ exhibiting the largest variability and ‘O’ displaying the lowest. Additional examination of the amplitude statistics indicates that the characters ‘D’, ‘H’, ‘L’, and ‘S’ have very elevated amplitudes, exceeding 13 millivolts. In contrast, the amplitudes of characters ‘A’, ‘B’, ‘C’, ‘O’, ‘R’, and ‘Y’ are low, measuring below 4 mV. According to the period statistics, the character ‘H’ has the highest period, while the character ‘O’ has the lowest time. The characters ‘D’, ‘E’, ‘G′, ‘H’, ‘L’, ‘P’, ‘Q’, ‘T’, ‘U’, ‘V’, ‘W’, and ‘X’ have periods that are relatively long, lasting more than 8000 s. Conversely, the characters ‘A’, ‘B’, ‘C’, ‘F’, ‘J’, ‘K’, ‘M’, ‘*N*’, ‘O’, ‘R’, ‘Y’, and ‘Z’ exhibit shorter periods, which are less than 2000 s. These findings indicate that when English alphabet characters are optically stimulated, they exhibit specific amplitude and period properties. The differences in the magnitude and duration of various features may have consequences for how characters are visually perceived and processed. Characters that have greater magnitudes and longer durations may be more readily distinguished or necessitate more time for processing in comparison to those with smaller magnitudes and shorter durations.

Figure 10a presents a contour plot of the optical recognition capacities of proteinoids toward the English alphabet. The contour plot represents the amplitude (mV) on the left *y*-axis and the period (s) on the right *y*-axis for each alphabet character along the *x*-axis. The contour fill colors correspond to the period, with black representing 0 s, green representing 5925 s, and red representing 11,850 s (1.185 × 10^4^ s). The amplitude values range from 1 to 2 mV. The plot visualizes the relationship between the amplitude and period for each character, revealing distinct patterns and variations in the optical recognition capacities of proteinoids. It can be observed that characters such as ‘D’, ‘H’, ‘L’, and ‘S’ exhibit higher amplitudes and longer periods, indicating enhanced optical recognition capacities. Conversely, characters like ‘A’, ‘B’, ‘C’, ‘O’, ‘R’, and ‘Y’ show lower amplitudes and shorter periods, suggesting reduced optical recognition capacities. Figure 10b presents a bubble plot of the optical recognition capacities of proteinoids toward the English alphabet. The bubble plot displays the amplitude (mV) on the *y*-axis and the alphabet characters on the *z*-axis. The size of the bubbles represents the period (seconds) of proteinoids for the optical recognition of each character. The bubble sizes are categorized into four ranges: 900–4550 s, 4550–8200 s, 8200–11,850 s, and 11,850 s and above. The color mapping of the bubbles further emphasizes the period ranges. The plot provides a visualization of the amplitude, period, and character relationships, highlighting the variations in the optical recognition capacities of proteinoids for different alphabet characters. Characters with larger bubbles, such as ‘D’, ‘H’, ‘L’, and ‘S’, indicate longer periods and enhanced optical recognition capacities. On the other hand, characters with smaller bubbles, such as ‘A’, ‘B’, ‘C’, ‘O’, ‘R’, and ‘Y’, suggest shorter periods and reduced optical recognition capacities. The contour plot and bubble plot in Figure 10 offer complementary perspectives on the optical recognition capacities of proteinoids toward the English alphabet. The contour plot focuses on the relationship between amplitude and period for each character, using color-coded contour fills to represent the period ranges. The bubble plot incorporates an additional dimension by representing the period as the size of the bubbles, allowing for a clear comparison of the optical recognition capacities across different characters. We examined the optical recognition responses of proteinoids that correspond to various English alphabet characters by stimulating them with light and measuring the potential (mV) over time (s). Figure 7 illustrates the characteristic potential vs time diagrams for nine representative characters: C, R, Y, E, P, U, T, Q, and H. The potential responses of the different letters exhibit significant variation, as illustrated in Figure 7. The amplitude of the responses varies from comparatively low values for characters such as C and R to higher values for characters such as E, P, and H. The duration of the responses also varies with certain letters exhibiting longer responses than others. For example, the response to the letter Y is more persistent than the response to the letter C. Furthermore, the response curves exhibited a wide range of morphologies, including asymmetric curves with distinct peaks (e.g., Q and H) and more symmetric and smooth profiles (e.g., U, T). Differences in the molecular structures and the conformational changes induced by light may account for these variations in the optical response characteristics of the proteinoids. The distinct response profiles of each proteinoid letter indicate that they hold distinctive optical recognition properties. These properties can be harnessed to develop proteinoid-based systems for alphabet character recognition.

### Boolean Gate Analysis of Proteinoid Optical Recognition of the English Alphabet

To analyze the optical recognition capabilities of proteinoids for the English alphabet characters, we employed Boolean gates as a means to model their behavior in response to optical stimuli. The amplitude and period data for each character were extracted from the ampData and periodData matrices, respectively. The data was then normalized to a range of 0 to 1 using min-max normalization, which is given by
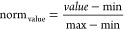
10where *value* is the original value, *min* is the minimum value in the data set, and *max* is the maximum value in the data set. To define the thresholds for the Boolean gates, we introduced threshold variables *T*_A_ for amplitude and *T*_P_ for period. These thresholds were set based on the normalized values of amplitude and period. The Boolean gates were defined using the following equations:

AND Gate:
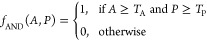
11OR Gate:
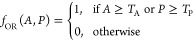
12NOT Gate (Amplitude):
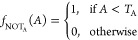
13NOT Gate (Period):
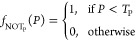
14NAND Gate:
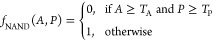
15NOR Gate:
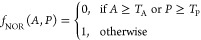
16

**Figure 11:**
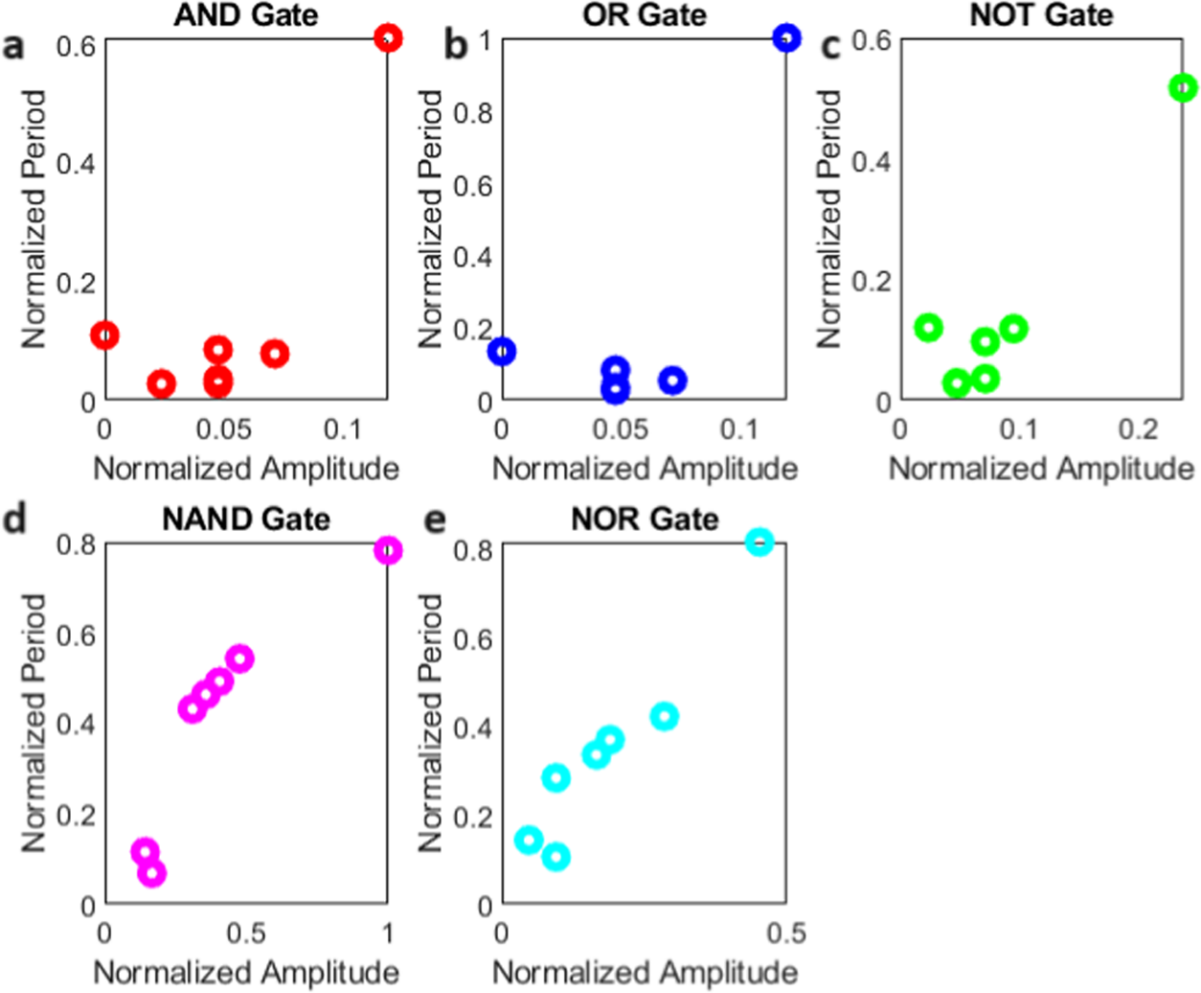
Analysis of proteinoid optical recognition for English alphabet symbols using Boolean gates. The figures depict the response of proteinoids to visual stimuli, simulated using various Boolean gates. Every data point in the plot represents a particular character, and its position is determined by its normalized amplitude and period values. The AND gate (a) depicts characters that have both amplitude and period values over their respective thresholds, whereas the OR gate (b) indicates characters that have either amplitude or period values that exceed the threshold. The NOT gates (c) demonstrate the negative correlation between amplitude and period, emphasizing features that fall below the thresholds. The NAND gate (d) and NOR gate (e) exhibit the opposite behavior of the AND and OR gates, respectively. The reason for the absence of all 26 characters in the plots is the normalization procedure, which converts the amplitude and period values to a range of 0 to 1. Characters that have comparable normalized values can potentially overlap or cluster in close proximity, leading to a reduced number of different data points. In addition, certain characters may exceed the depicted range due to their exceptionally high amplitude or period values compared to the remainder of the sample. The examination of Boolean gates offers valuable insights into the optical identification capacities of proteinoids and their reactions to optical stimuli, which are determined by certain thresholds.

In these equations, *A* represents the normalized amplitude value, *P* represents the normalized period value, and *T*_A_ and *T*_P_ are the corresponding thresholds. Figure 11 shows the plots of the Boolean gates based on the extracted amplitude and period data for the English alphabet characters. Each plot represents a different Boolean gate, with the normalized amplitude on the *x*-axis and the normalized period on the *y*-axis. The AND gate plot (Figure 11a) shows the behavior of the proteinoids when both the amplitude and period values are above their respective thresholds. Characters that satisfy this condition are clustered toward the upper-right corner of the plot. The OR gate plot (Figure 11b) represents the behavior when either the amplitude or period value exceeds its threshold. Characters that meet this criterion are scattered along the upper-right and lower-right regions of the plot. The NOT gate plots (Figure 11c) illustrate the inverse relationship between the amplitude and period. Characters with amplitude or period values below their respective thresholds are mapped to the upper-left corner of the plots. The NAND gate plot (Figure 11d) shows the inverse of the AND gate behavior, where characters that do not satisfy both amplitude and period thresholds are clustered toward the lower-left corner. Finally, the NOR gate plot (Figure 11e) represents the inverse of the OR gate, with characters that have neither amplitude nor period values exceeding their thresholds mapped to the lower-left region. By adjusting the threshold values *T*_A_ and *T*_P_, we can control the behavior of the Boolean gates and determine which characters satisfy the conditions based on their normalized amplitude and period values. This analysis provides insights into the optical recognition capabilities of proteinoids for different English alphabet characters and helps in understanding their response to optical stimuli in terms of Boolean logic.

Table 3 presents the analysis results of the Boolean gates for the optical recognition of the English alphabet using proteinoids. The table provides a summary of the number of characters that fulfill the requirements for each Boolean gate, along with a list of the specific characters that fit those requirements. Table 3 demonstrates that none of the English alphabet characters meet the criteria for both high amplitude and long period simultaneously (as required by the AND gate) or for either high amplitude or long period (as required by the OR gate) according to the specified thresholds. Conversely, the NOT gate requirement is met with all 26 characters of the English alphabet. This implies that all characteristics exhibit either a small magnitude or a brief duration, both of which are below the prescribed thresholds. The characters that fulfill the criteria of the NOT gate are A, B, C, D, E, F, G, H, I, J, K, L, M, N, O, P, Q, R, S, T, U, V, W, X, Y, and Z. All 26 characters of the English alphabet satisfy the criteria of both the NAND gate and NOR gate. The NAND gate is the logical complement of the AND gate, meaning that it outputs a high signal only when none of the input signals meet both the high amplitude and long period criteria at the same time. The NOR gate is the logical complement of the OR gate, indicating that none of the inputs have a large amplitude or lengthy period according to the specified criteria. These studies offer valuable information about the ability of proteinoids to recognize English alphabet symbols optically. If there are no characters that meet the requirements of the AND gate and OR gate, then it means that the proteinoids do not show a significant response to optical stimuli in terms of both amplitude and period, either together or separately. Nevertheless, the observation that all characters meet the criteria for the NOT gate, NAND gate, and NOR gate suggests that the proteinoids consistently respond to low-amplitude and short-duration visual inputs across all characters. Boolean gate analysis facilitates the understanding of proteinoid behavior in relation to optical stimuli and their potential for optical character recognition.

**Table 3:** Boolean Gate Analysis Results

gate	count	characters
AND Gate	0	
OR Gate	0	
NOT Gate	26	A, B, C, D, E, F, G, H, I, J, K, L, M, N, O, P, Q, R, S, T, U, V, W, X, Y, Z
NAND Gate	26	A, B, C, D, E, F, G, H, I, J, K, L, M, N, O, P, Q, R, S, T, U, V, W, X, Y, Z
NOR Gate	26	A, B, C, D, E, F, G, H, I, J, K, L, M, N, O, P, Q, R, S, T, U, V, W, X, Y, Z

### Spontaneous Spiking of Proteinoids

**Figure 12:**
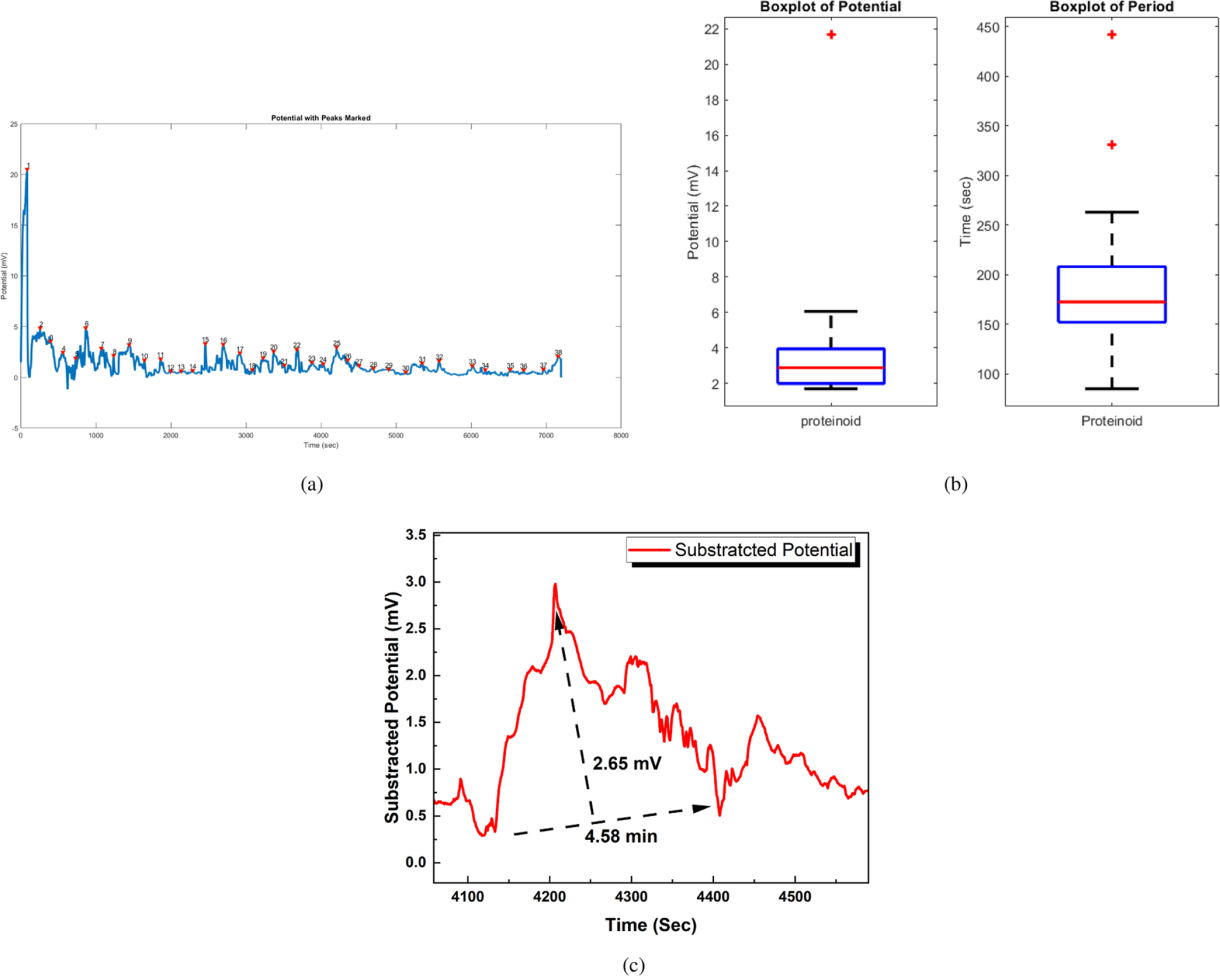
Electrical activity and statistical analysis of proteinoid microspheres. (a) Normalized potential (mV) vs time (s) plot highlighting the characteristic spikes. Each numbered point corresponds to a specific spike in the signal. (b) Box plots illustrating the statistical distributions of the spike amplitudes (potential) and periods. The potential box plot shows quartiles of 1.99, 2.88, and 3.95 mV, a mean of 3.56 mV, a maximum of 21.68 mV, a minimum of 1.70 mV, and a standard deviation of 3.23 mV. The period box plot displays quartiles of 152.00, 172.50, and 208.00 s, a mean of 188.39 s, a maximum of 442.00 s, a minimum of 85.00 s, and a standard deviation of 60.77 s. (c) Enlarged view of a representative spike with an amplitude of 2.65 mV and a period of 4.58 min (274.8 s), providing a detailed examination of the spike characteristics.

**Figure 13:**
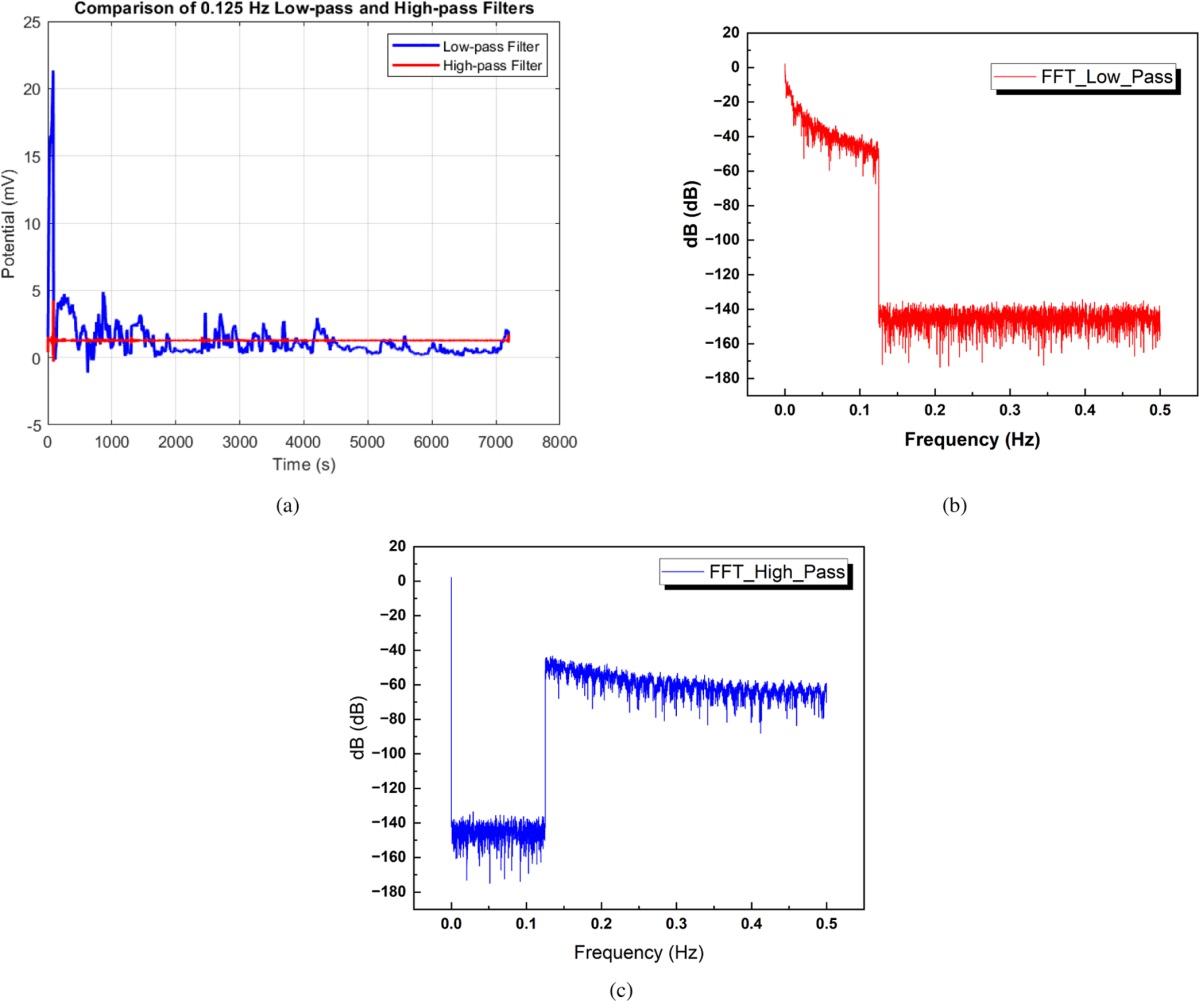
Comparative analysis of low-pass and high-pass filters applied to the electrical activity of proteinoid microspheres. (a) Potential (mV) vs time (s) plot illustrating the filtered signals, with the low-pass filter in blue and the high-pass filter in red. The low-pass filter (*N* = 7197, mean = 1.28237 mV, SD = 0.0611 mV, sum = 9229.23071 mV, min = −0.29314 mV, median = 1.28248 mV, max = 4.26473 mV) removes high-frequency components, resulting in a smoother signal, while the high-pass filter (*N* = 7197, mean = 1.28237 mV, SD = 1.81627 mV, sum = 9229.23047 mV, min = −1.11956 mV, median = 0.78598 mV, max = 21.33927 mV) eliminates low-frequency components, emphasizing rapid changes in the signal. (b) Frequency domain representation of the low-pass filtered signal (*N* = 3599, mean = −81.39973 dB, SD = 37.61018 dB, sum = −292957.641 dB, min = −174.88367 dB, median = −62.75598 dB, max = 2.16028 dB), with the *y*-axis in decibels (dB). The low-pass filter attenuates high frequencies, allowing low frequencies to pass through. (c) Frequency domain representation of the high-pass filtered signal (*N* = 3599, mean = −118.50095 dB, SD = 47.48328 dB, sum = −426484.92036 dB, min = −173.63782 dB, median = −142.78386 dB, max = 2.16028 dB), with the *y*-axis in decibels (dB). The high-pass filter attenuates low frequencies, allowing high frequencies to pass through. These results demonstrate the effectiveness of the low-pass and high-pass filters in separating the low-frequency and high-frequency components of the electrical activity of proteinoid microspheres. The statistical measures, such as mean, standard deviation, minimum, median, and maximum, characterize the overall behavior and variability of the filtered signals in both the time domain and frequency domain, facilitating the comparison and interpretation of the filtered signals and enabling a deep understanding of the electrical activity of the proteinoid microspheres.

The electrical activity and statistical analysis of proteinoid microspheres are presented in Figure 12. The normalized potential (mV) vs time (s) plot in Figure 12a highlights the characteristic spikes exhibited by the microspheres, with each numbered point corresponding to a specific spike in the signal. To gain insights into the statistical properties of these spikes, Figure 12b displays box plots illustrating the distributions of spike amplitudes (potential) and periods. The potential box plot reveals quartiles of 1.99, 2.88, and 3.95 mV, a mean of 3.56 mV, a maximum of 21.68 mV, a minimum of 1.70 mV, and a standard deviation of 3.23 mV. Similarly, the period box plot shows quartiles of 152.00, 172.50, and 208.00 s, a mean of 188.39 s, a maximum of 442.00 s, a minimum of 85.00 s, and a standard deviation of 60.77 s. These statistical measures provide an in-depth analysis of the spike amplitudes and periods, enabling a better understanding of the electrical behavior of the proteinoid microspheres. To further examine the morphology of individual spikes, Figure 12c presents an enlarged view of a representative spike with an amplitude of 2.65 mV and a period of 4.58 min (274.8 s). This detailed view allows for a closer inspection of the spike characteristics and facilitates a deeper analysis of the electrical activity at the single-spike level. To further investigate the frequency components of the electrical activity, Figure 13 presents a comparative analysis of low-pass and high-pass filters applied to the proteinoid microsphere signals. Figure 13a displays the potential (mV) vs time (s) plot of the filtered signals, with the low-pass filter in blue and the high-pass filter in red. The low-pass filter, characterized by N = 7197, mean = 1.28237 mV, SD = 0.0611 mV, sum = 9229.23071 mV, min = −0.29314 mV, median = 1.28248 mV, and max = 4.26473 mV, removes high-frequency components, resulting in a smoother signal. In contrast, the high-pass filter, with N = 7197, mean = 1.28237 mV, SD = 1.81627 mV, sum = 9229.23047 mV, min = −1.11956 mV, median = 0.78598 mV, and max = 21.33927 mV, eliminates low-frequency components, emphasizing rapid changes in the signal. The frequency domain representations of the low-pass and high-pass filtered signals are shown in Figure 13b,c, respectively, with the *y*-axis in decibels (dB). The low-pass filter attenuates high frequencies, allowing low frequencies to pass through, while the high-pass filter attenuates low frequencies, allowing high frequencies to pass through. The statistical measures provided for each filtered signal, including mean, standard deviation, minimum, median, and maximum, characterize the overall behavior and variability of the signals in both the time domain and frequency domain. These results demonstrate the effectiveness of the low-pass and high-pass filters in separating the low-frequency and high-frequency components of the electrical activity of proteinoid microspheres, facilitating a comprehensive analysis of the signal characteristics. The combination of the electrical activity analysis in Figure 12 and the frequency domain analysis using low-pass and high-pass filters in Figure 13 provides a multifaceted understanding of the proteinoid microsphere signals. The statistical measures and visual representations in both figures enable a thorough characterization of the spike properties, frequency components, and overall behavior of the electrical activity exhibited by the microspheres. These results contribute to a deeper understanding of the complex electrical dynamics of proteinoid microspheres and their potential implications in various applications.

**Figure 14:**
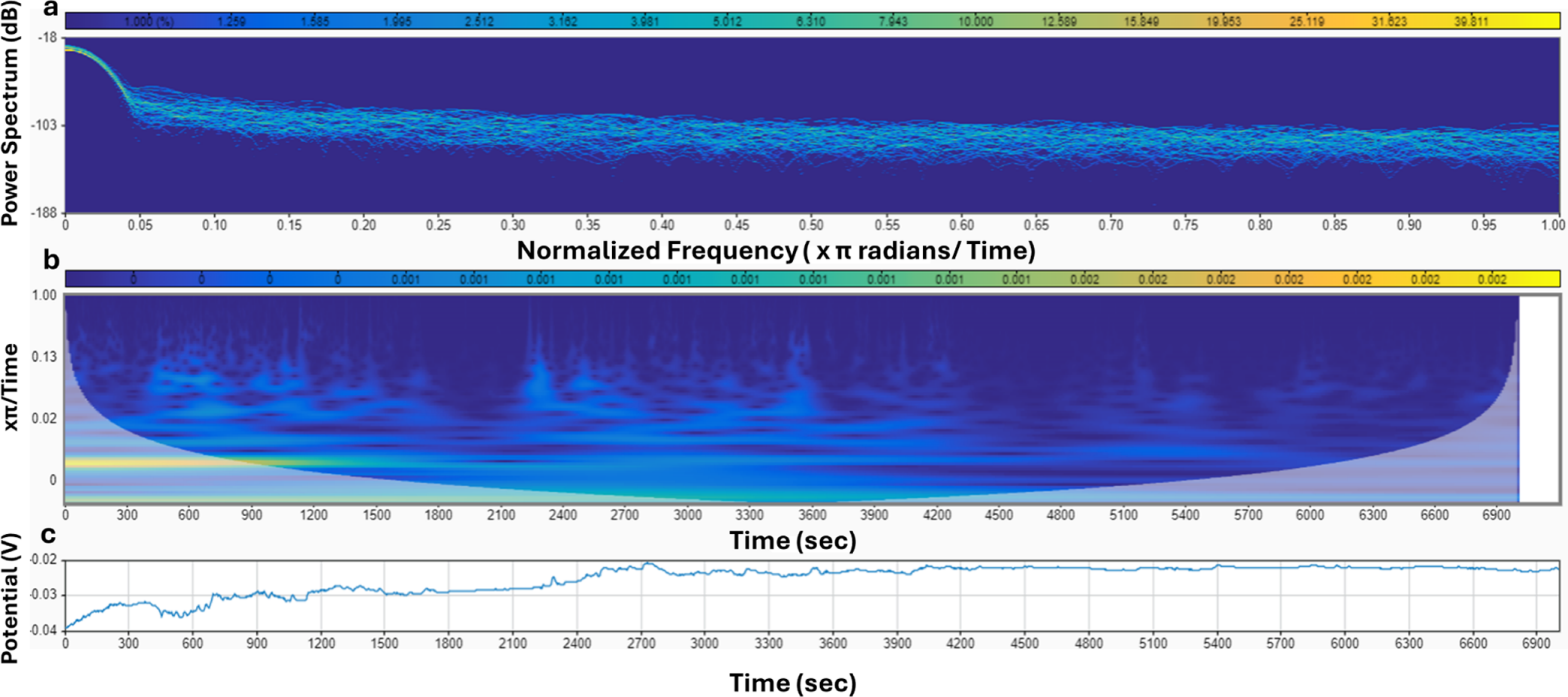
Spectral analysis of the proteinoid l-Glu:l-Asp:l-Phe signal. (a) Persistent spectrum of the proteinoid signal, with the *y*-axis representing the power spectrum (dB) and the *x*-axis representing the normalized frequency (Hz). (b) Scalogram of the real signal, displaying time (s) on the *x*-axis and xπ/time on the *y*-axis. (c) Proteinoid signal, with potential (V) plotted against time (s). The amplitude shows quartiles of 1.99, 2.88, and 3.95 mV, a mean of 3.56 mV, a maximum of 21.68 mV, a minimum of 1.70 mV, and a standard deviation of 3.23 mV. The period displays quartiles of 152.00, 172.50, and 208.00 s, a mean of 188.39 s, a maximum of 442.00 s, a minimum of 85.00 s, and a standard deviation of 60.77 s.

The persistent spectrum in Figure 14a displays the prevailing frequencies found in the proteinoid l-Glu:l-Asp:l-Phe signal. The peak with the maximum amplitude in the power spectrum represents the frequency component that stands out the most, while lower peaks indicate the existence of other periodic components in the signal. The scalogram seen in Figure 14b offers a visual depiction of the signal’s time-frequency characteristics, enabling the detection of changes in frequency across time. The scalogram visually represents the temporal evolution of dominant frequencies, with darker areas representing greater signal energies at various time points and frequencies. The persistent spectrum and scalogram together allow for a thorough examination of the spectral features and temporal changes of the proteinoid signal.

## Discussion

The electrical properties of proteinoids, including amplitude and period, are tightly connected to their distinct natures and composition. Proteinoids are formed through the thermal polycondensation of amino acids, which leads to the formation of a complex network of peptide bonds and interactions between side chains. The precise configuration of amino acids and the presence of charged residues play roles with regard to the electrical properties of proteinoids. The variations in amplitude and period found in various proteinoid samples can be ascribed to differences in their molecular structure and the corresponding distribution of different amino acids. The electrical characteristics of proteinoids have substantial implications for their use in visual recognition systems. Proteinoids possess the capacity to produce unique electrical impulses when exposed to light-based stimuli, rendering them well suited for the encoding and manipulation of information. The fluctuations in magnitude and duration can be utilized to create robust and dependable optical character recognition (OCR) systems. By using the unique electrical characteristics of proteinoids, it is feasible to create OCR systems that are exceptionally sensitive and specific, enabling accurate identification and classification of various characters. Proteinoids have distinct signal processing characteristics when compared to other materials commonly employed in OCR systems, such as silicon-based devices or organic semiconductors. Proteinoids have exceptional sensitivity and selectivity when exposed to visual stimuli, allowing them to precisely differentiate between various features. Proteinoids’ biocompatibility and biodegradability make them a compelling substitute for traditional materials. Furthermore, the capacity of proteinoids to spontaneously self-assemble and create durable layers amplifies their potential for incorporation into the OCR systems.

Multiple variables can influence the precision of the proteinoid-based optical character recognition (OCR) system. The performance of the system is heavily influenced by the quality of the input signal, including factors such as the resolution and contrast of the characters. Excessive noise and distortions in the input signal can result in inaccuracies in the character recognition. The characteristics of the proteinoid film, such as its thickness, homogeneity, and stability, also impact the accuracy of recognition. In addition, it is necessary to optimize system characteristics, such as illumination conditions, signal amplification, and signal processing methods, in order to achieve high recognition rates. In order to enhance the precision of recognition, a range of methods can be used. Preprocessing methods, such as enhancing the image and reducing noise, can be used to enhance the quality of the incoming signal. Optimizing the characteristics of the proteinoid film, such as its composition and deposition, can facilitate the production of films with favorable electrical properties. Moreover, sophisticated signal processing algorithms, such as machine learning approaches, can be employed to improve the performance of character recognition. Using feedback mechanisms and adaptive learning methodologies can enhance recognition accuracy over time. Although the proteinoid-based OCR system has its advantages, it also encounters specific constraints and obstacles. Further research and improvement are required to examine and enhance the long-term stability and durability of proteinoid films. The system’s response time and speed may be comparatively slower than that of traditional electrical devices, which can restrict its use in high-speed, high-speed, and low-temperature OCR systems. Furthermore, it is necessary to consider the ability to scale up and the cost-effectiveness of proteinoid production and device fabrication procedures in order to make them feasible for actual use.

The results given in this work have important consequences for the advancement of sophisticated spectral OCR systems. The experimental study of the optical recognition features of proteinoids presents new opportunities for developing bioinspired and biomaterial-based recognition systems. Proteinoids provide distinctive electrical characteristics and signal processing capabilities that can be utilized to develop OCR systems that are exceptionally sensitive and discerning, resulting in enhanced performance and dependability. In addition to the use of OCR, proteinoids have the capacity to be utilized in diverse areas such as pattern recognition, machine learning, and unconventional computing. Proteinoids possess the capacity to manipulate and store data, making them valuable for the development of innovative computer systems and algorithms. Proteinoids can be used for several applications, such as image recognition, speech recognition, and biosignal processing. Due to their biocompatibility and ability to self-assemble, these materials show great potential for being used in conjunction with living systems and for creating biohybrid technologies. In order to enhance and optimize the proteinoid-based OCR system, it is necessary to focus on certain specific areas. By adjusting the composition and production conditions of proteinoids, it is possible to optimize their electrical characteristics and improve their ability to recognize certain analytes. Examining the use of various combinations of amino acids and investigating the consequences of chemical changes can result in the production of proteinoids with customized traits for certain purposes.

**Figure 15:**
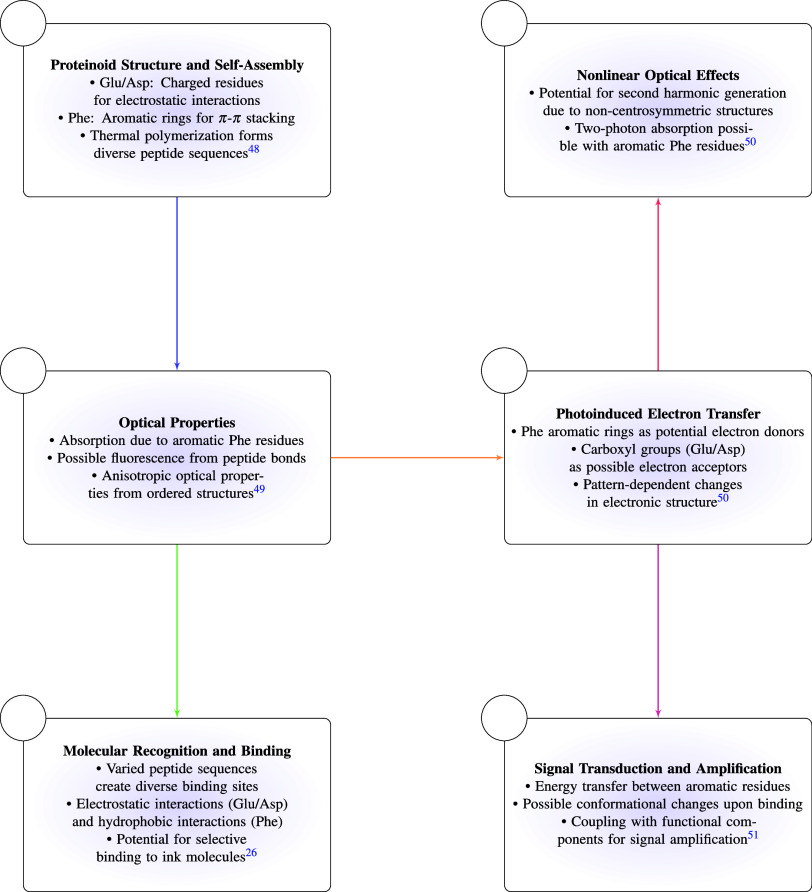
Molecular basis for optical recognition of the English alphabet using the proteinoid l-glutamic acid:l-aspartic acid:l-phenylalanine. The unique combination of these amino acids provides a balance of charged (Glu/Asp) and aromatic (Phe) residues, enabling diverse interactions and optical properties crucial for the OCR application.

The OCR application of our proteinoid material significantly depends on the selection of l-glutamic acid, l-aspartic acid, and l-phenylalanine. This blend offers a special balance of charged (Glu/Asp) and aromatic (Phe) residues, therefore allowing different molecular interactions and optical characteristics. The thermal polymerization method produces a complex mixture of peptides with different sequences, therefore producing a material with several recognition sites and optical sensing capacity. Specifically, although the aromatic rings of phenylalanine help π – π stacking and possible photoinduced electron transfer processes, the charged residues enable electrostatic interactions with ink molecules. Our OCR technology is based on pattern-dependent modifications in optical characteristics made possible by this molecular diversity. We have now extended our prior work^26^ on the effectiveness of this proteinoid composition in implementing unconventional computing paradigms to OCR applications. The proposed mechanism for optical recognition of the English alphabet using the proteinoid l-glutamic acid:l-aspartic acid:l-phenylalanine, as illustrated in Figure 15, involves several key steps. Molecular recognition,^40,41^ photoinduced electron transfer,^42,43^ and nonlinear optical effects^44^ play crucial roles in the optical recognition of the English alphabet using the proteinoid l-glutamic acid:l-aspartic acid:l-phenylalanine. The molecular recognition and binding process can be modeled using the Hill equation, which describes the cooperative binding of ligands to a macromolecule:
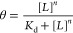
17where θ is the fraction of binding sites occupied, [*L*] is the ligand concentration, *K*_d_ is the dissociation constant, and *n* is the Hill coefficient. This equation provides insights into the binding affinity and cooperativity of the proteinoid–ligand interactions. The photoinduced electron transfer process, which contributes to the optical recognition mechanism, can be described using the Marcus theory of electron transfer.^45,46^ This theory relates the rate of electron transfer (*k*_ET_) to the reorganization energy (λ) and the driving force (Δ*G*^0^):
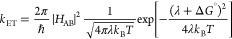
18where *H*_AB_ is the electronic coupling between the donor and acceptor, *k*_B_ is the Boltzmann constant, and *T* is the temperature. This equation helps us to understand the factors influencing the electron transfer process and its role in the optical recognition mechanism. Nonlinear optical effects, such as second harmonic generation, are also involved in the optical recognition process and can be described using the nonlinear polarization equation:
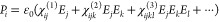
19where *P*_*i*_ is the induced polarization, ε_0_ is the permittivity of free space, χ^*n*^ are the *n*-th order susceptibility tensors, and *E*_*j*_, *E*_*k*_, and *E*_*l*_ are the electric field components.

Future research should prioritize improving the range of characters recognized by the proteinoid-based OCR system. Expanding the investigation into the system’s ability to recognize a broader range of characters, such as numerals, special characters, and multilingual scripts, would increase the system’s usefulness. By the incorporation of other recognition technologies, such as machine learning algorithms and computer vision techniques, the OCR system can be significantly improved in terms of its reliability and precision. Furthermore, investigating the potential of proteinoids for immediate and dynamic character recognition can unveil novel opportunities for interactive and adaptable OCR systems.^47−50^

This study expands on the idea of proteinoid microspheres, which are an important advancement in the progression toward complex structures made from basic amino acid polymers. Proteinoid microspheres can be easily formed under various conditions, such as temperature fluctuations or the addition of water to molten proteinoid, as explained by Fox.^51^ These microspheres display a variety of characteristics and patterns that are crucial for evolution, including stable and selectively permeable boundaries, catalytic activities, movement, compartmentalization, and the capacity to communicate and reproduce. The use of proteinoids and microspheres as components of systems that facilitate prebiologically significant phenomena further emphasizes their potential as functional materials. For instance, proteinoids that are abundant in lysine have been demonstrated to enhance the production of aminoacyl adenylates from amino acids and ATP. These compounds act as both building blocks and energy sources for the synthesis of polyamino acids. Furthermore, the correlation between proteinoids high in lysine and homopolynucleotides has been examined as a representation of early ribosomes, facilitating the creation of 1,N6-etheno derivatives of the adenine ring.

The use of proteinoids for visual identification of the English alphabet, as demonstrated in this study, introduces a new and innovative application of these materials, expanding their capabilities beyond prebiological systems. We have shown that proteinoids possess distinctive features that make them suitable for advanced technological applications, including character recognition and optical computing.

The development of proteinoid-based optical character recognition (OCR) systems represents a significant advancement in biocompatible and environmentally sustainable computing technologies. Proteinoid-based alternatives have unique advantages over conventional electronic OCR systems in biomedical applications that prioritize biocompatibility. For example, these systems have the potential to be integrated into smart contact lenses and implantable medical devices. This would allow for real-time text recognition and information processing within the body, minimizing the risk of negative biological responses. In addition, the biodegradable properties of proteinoids, along with their production from renewable resources, make this technology a more sustainable option compared with conventional electronic OCR systems. This is in line with the increasing global need for environmentally friendly computing solutions. It has the potential to reduce electronic waste caused by current OCR technologies while still being effective. Proteinoids have inherent properties that offer numerous advantages beyond their biocompatibility and sustainability. These structures have the ability to self-assemble, indicating their potential for adaptive learning in demanding environments where conventional electronic systems may not be effective, such as those with extreme temperatures or electromagnetic interference. The adaptability of the technologies of OCR has the potential to greatly increase their operational range. In addition, OCR systems based on proteinoids have the potential to be fundamental components in advanced bioinspired computing structures. This could lead to the development of innovative approaches in chemical intelligence and machine learning that closely mimic the information processing mechanisms found in biological systems. The small size of proteinoid structures allows for the possibility of highly compact OCR systems, which could potentially revolutionize information processing by operating at scales that are currently beyond the reach of conventional electronic systems. This molecular-scale information processing capability has the potential to bring about significant advancements in various fields including nanotechnology and advanced data analytics. It promises remarkable levels of miniaturization and efficiency in tasks such as character recognition and data processing.

We used various methods to analyze our proteinoid material. This ensured reproducibility and confirmed its structure–function relationships. UV experiments showed consistent absorption patterns across batches.^29^ FT-IR spectra indicated that peptide bonds formed.^31^ We acknowledge that additional analytical methods would provide deeper insights into the material’s molecular architecture. Future studies should use techniques such as circular dichroism (CD) spectroscopy to analyze secondary structures, nuclear magnetic resonance (NMR) spectroscopy to determine sequence distributions, and mass spectrometry to evaluate molecular weight distributions. Adding these characterizations would help create better structure–property relationships. It would also improve the optical recognition of proteinoid-based systems. Our proteinoid-based OCR system works by a unique interplay of charged (Glu/Asp) and aromatic (Phe) residues. This creates many recognition sites through electrostatic and π–π stacking interactions.^15,22^ The thermal polymerization process creates a mix of peptide sequences. This results in materials with different binding sites and optical properties.^16,17^ The diverse molecules allow for changes in the optical properties. These depend on specific patterns. This enables character recognition. Proteinoids can self-organize.^21^ This allows them to make stable microspheres that reliably react to light. Our SEM analysis supports this. It shows size distributions of 500 nm to 3 μm across various synthesis batches. The use of proteinoids in OCR apps is a big step in bioinspired computing systems.^33^ Unlike traditional electronic OCR systems, proteinoid-based recognition uses natural characteristics. It relies on their photoinduced electron transfer mechanisms.^8,30^ Our studies show that the electrical responses are very specific to certain character patterns. The proteinoid microspheres can, with their unique mechanisms, distinguish between different optical inputs.^25^ A deeper look at the molecular mechanisms in this pattern recognition process would improve our understanding of the system’s strengths and weaknesses. Future work should focus on creating structure–activity relationships. Additionally, establishing more rigorous performance metrics for the comparison of proteinoid-based OCR systems with traditional technologies is essential. This work demonstrates the diversity and adaptability of proteinoids, highlighting their capacity to connect prebiological systems with modern technology applications. The effective demonstration of proteinoids in optical character recognition paves the way for further exploration and advancement in the realm of bioinspired materials and computers.

## Conclusions

The use of proteinoids for the optical recognition of the English alphabet signifies a noteworthy progression in the domain of bioinspired materials and computing. This study showcases the multifunctionality and flexibility of proteinoids, highlighting their potential as effective materials for advanced technological applications. We have devised a novel technique for optical character identification by using the distinctive characteristics of proteinoids, such as their capacity to produce stable and selectively permeable microspheres. The findings of this study not only emphasize the effectiveness of proteinoids in carrying out complex tasks such as character identification but also emphasize the importance of exploring the functional abilities of these materials beyond their significance in prebiological phenomena. As research in this subject advances, proteinoids are expected to be used in a wider range of applications including information processing, sensing, and computing. This work provides a basis for future research on the advancement of bioinspired materials and systems. It sets the stage for the development of more sophisticated and effective technologies that rely on the concepts of self-organization and evolutionary continuity. Proteinoids have the potential to provide sustainable, flexible, and intelligent solutions to challenging problems by connecting prebiological systems with modern technological applications.

## Data Availability

The data for this paper is available at the following link: https://zenodo.org/records/12522410.

## Supporting Information Available

Illustration of the energy optimization of the L-Glu:L-Asp:L-Phe peptide (Figure S1) and energy components and their corresponding values (Table S1) (PDF)
