# Supplementary material for: Optical Recognition of the English Alphabet Using Proteinoids
Source: ACS Omega. 2024 Dec 17;9(52):51098–119. doi: 10.1021/acsomega.4c06401 (PMC11696383; doi:10.1021/acsomega.4c06401)
Supplement: Supplementary file 1 — ao4c06401_si_001.pdf [file ao4c06401_si_001.pdf]

Supplementary Material

Optical Recognition of the English Alphabet Using Proteinoids

Panagiotis Mougkogiannis[1,*] and Andrew Adamatzky[1]

[1]Unconventional Computing Laboratory, University of the West of England, Bristol, UK

Email: Panagiotis.Mougkogiannis@uwe.ac.uk

Exploring the Dynamic Interactions of L-Glu:L-Asp:L-Phe: A Molecular Simulation Study

The L-Glu:L-Asp:L-Phe peptide was subjected to energy optimisation using MM2 minimization in order to analyse its structural stability and the impacts of different energy components. Figure S1 provides a detailed depiction of the outcomes achieved through energy optimisation. Figure S1(a) illustrates the molecular structure of the optimised L-Glu:L-Asp:L-Phe peptide, emphasising its three-dimensional conformation and the spatial organisation of the amino acid residues. The peptide's chemical structure is depicted in Figure S1(b), exhibiting the sequential arrangement of the amino acids and the peptide bonds that link them. The distinctive chemical characteristics of the side chains of glutamic acid (Glu), aspartic acid (Asp), and phenylalanine (Phe) residues are highlighted. The energy optimisation findings of the L-Glu:L-Asp:L-Phe peptide are displayed in Figure S1(c) and Table S1. The bar graph depicted in Figure S1(c) illustrates the individual contributions of several energy components to the overall energy of the optimised peptide structure. The energy values, measured in kcal/mol, consist of the following components: stretching (1.2400), bending (6.5174), stretch-bending (0.2652), torsion (-9.0748), non-1,4 van der Waals interactions (-25.5240), 1,4 van der Waals interactions (14.5482), and dipole-dipole interactions (-9.7099). Table S1 presents an in-depth analysis of the energy components and their respective values derived from the MM2 minimization process. The optimised peptide has a total energy of -21.7378 kcal/mol, indicating a stable conformation. The primary factors that contribute to the stability of the system are the non-1,4 van der Waals contacts and the torsional energy. Conversely, the stability is opposed by the 1,4 van der Waals interactions and the bending energy. The interplay between the stretch-bend coupling and the dipole-dipole interactions plays a significant role in enhancing the overall stability of the proteinoid structure. The energy decomposition analysis, depicted in Figure S1(c) and Table S1, offers valuable insights into the relative significance of various energy components in stabilising the peptide structure. The integration of the molecular representation, chemical structure, and energy optimisation findings provides a thorough comprehension of the structural and energetic characteristics of the L-Glu:L-Asp:L-Phe peptide. The results indicate that the L-Glu:L-Asp:L-Phe peptide has been successfully optimised for energy using MM2 minimization. This optimisation has shown the peptide's stable conformation and the interaction between different energy components that contribute to its structural stability.

In addition to the L-Glu:L-Asp:L-Phe peptide, Table S1 also presents the energy optimization results for 3-peptides, 5-peptides, and 15-peptides sequences. The 3-peptides have a total energy of -124.2770 kcal/mol, with significant contributions from non-1,4 van der Waals interactions and dipole-dipole interactions. The 5-peptides exhibit a total energy of -231.1312 kcal/mol, with non-1,4 van der Waals interactions and dipole-dipole interactions being the main stabilizing factors. The 15-peptides show the lowest total energy of -792.9097 kcal/mol, indi-

cating a highly stable structure. The energy decomposition analysis for the 15-peptides reveals the dominant role of non-1,4 van der Waals interactions, followed by dipole-dipole interactions and torsional energy in stabilizing the structure. Across all peptide sequences, the 1,4 van der Waals interactions and bending energy consistently oppose the stability. The energy decomposition analysis, depicted in Figure S1(c) and Table S1, offers valuable insights into the relative significance of various energy components in stabilising the peptide structures. The integration of the molecular representation, chemical structure, and energy optimisation findings provides a thorough comprehension of the structural and energetic characteristics of the studied peptides. The results indicate that all the investigated peptide sequences, including L-Glu:L-Asp:L-Phe, 3-peptides, 5-peptides, and 15-peptides, have been successfully optimised for energy using MM2 minimization. This optimisation has shown the stable conformations of the peptides and the interaction between different energy components that contribute to their structural stability. The increasing stability observed with longer peptide sequences highlights the importance of peptide length in determining the overall structural stability of proteinoid systems.

It is crucial to recognise that the calculations shown in Figure S1 and Table S1, which assume a Glu-Asp-Phe repeating pattern, represent an idealised model of the proteinoid structure. The proteinoid material is likely to have a diverse composition, potentially with different ratios of amino acids and even segments of homopolymers. Although there is a limitation, our model offers valuable insights into the energetics of the system. Firstly, it acts as a fundamental model system, providing a basis for understanding the potential energy contributions and interactions within the proteinoid material. The energy decomposition analysis uncovers the significance of different interactions in stabilising peptide structures, even in more diverse compositions. In addition, our calculations on 3-peptides, 5-peptides, and 15-peptides showcase the scalability of these interactions, offering valuable insights into the energetic behaviour of larger and more complex structures. Significantly, these calculations provide a standard for comparing experimental results. Deviation from the idealised model can offer valuable insights into the actual composition and structural arrangements of the synthesised proteinoid material. In future research, a broader range of computational models will be investigated. This will involve examining different amino acid ratios and sequence arrangements to more accurately capture the potential heterogeneity of the material. In addition, we intend to perform additional experimental characterisation techniques, such as mass spectrometry or chromatography, to obtain a greater knowledge of the composition and distribution of peptide sequences in our proteinoid material.

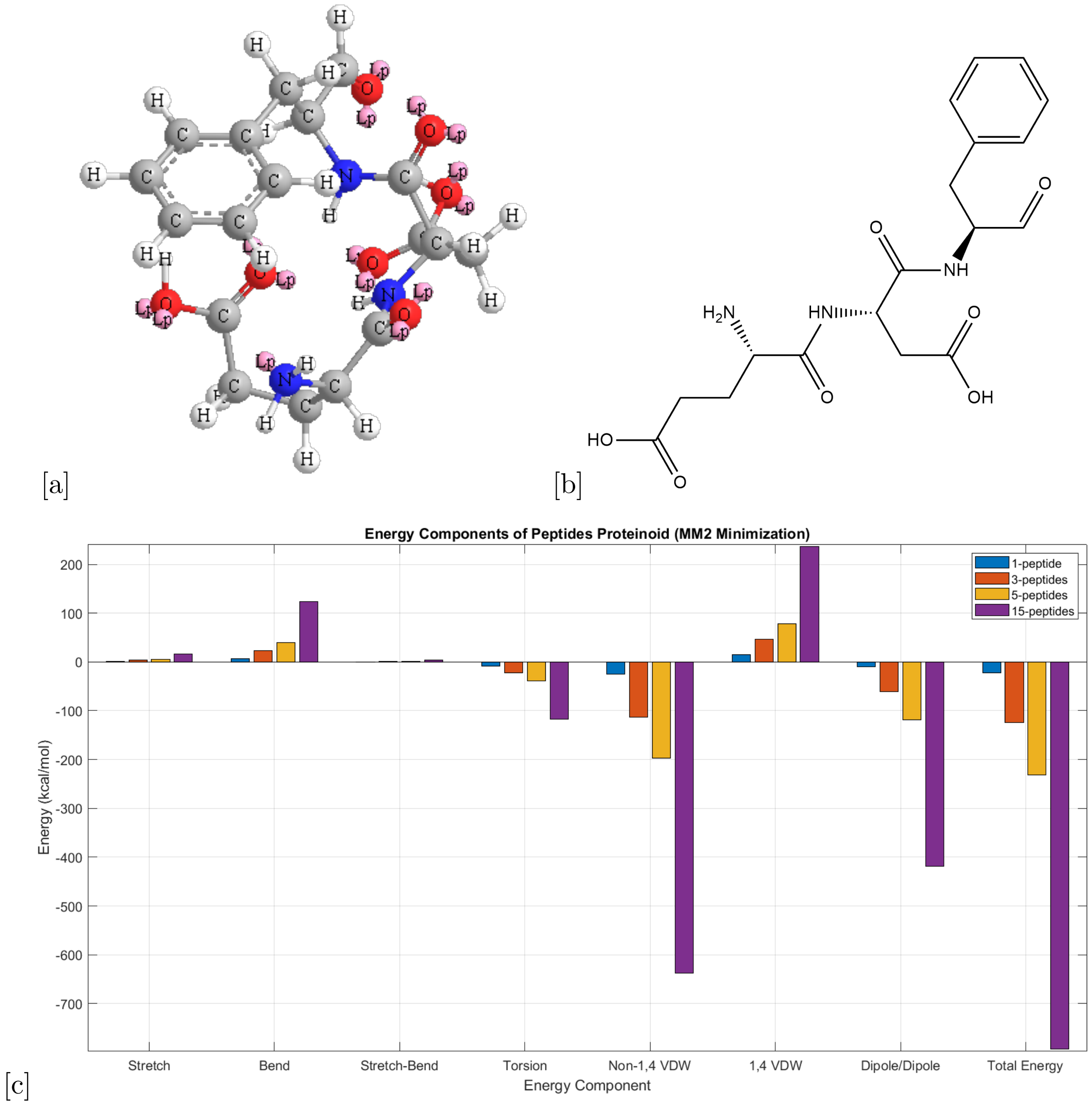

Figure S1: Illustration of the energy optimisation of the L-Glu:L-Asp:L-Phe peptide. (a) Molecular depiction of the optimised structure of the L-Glu:L-Asp:L-Phe peptide. The visualisation illustrates the three-dimensional structure of the peptide following energy minimization, emphasising the spatial organisation of the amino acid residues. (b) Chemical structure of the L-Glu:L-Asp:L-Phe peptide. The schematic representation illustrates arrangement of amino acids and the chemical bonds that link them, known as peptide bonds. The different chemical characteristics of the side chains of glutamic acid (Glu), aspartic acid (Asp), and phenylalanine (Phe) residues are highlighted. (c) Energy optimization results of the 3-peptides, 5-peptides, and 15-peptides. The energy values, measured in kcal/mol, include stretch, bend, stretch-bend, torsion, non-1,4 van der Waals interactions, 1,4 van der Waals interactions, and dipole-dipole interactions. The optimized 3-peptides, 5-peptides, and 15-peptides have total energies of -124.2770 kcal/mol, -231.1312 kcal/mol, and -792.9097 kcal/mol, respectively, indicating stable conformations. The energy decomposition analysis reveals the significant contributions of non-1,4 van der Waals interactions and dipole-dipole interactions to the stability of the peptide structures, while the 1,4 van der Waals interactions and bending energy oppose the stability. The interplay of these energy components provides insights into the structural and energetic properties of the peptides, with the 15-peptides exhibiting the highest stability among the studied systems.

Table S1: Energy components and their corresponding values obtained from the MM2 minimization of the L-Glu:L-Asp:L-Phe proteinoid and additional peptide sequences (3-peptides, 5-peptides, and 15-peptides). The total energy of −21.7378 kcal/mol for the L-Glu:L-Asp:L-Phe proteinoid indicates a stable minimized structure. The major contributors to its stability are the non-1,4 van der Waals interactions and the torsional energy, while the 1,4 van der Waals interactions and the bending energy oppose the stability. The stretch-bend coupling and the dipole-dipole interactions also contribute to the overall stability of the proteinoid structure. For the 3-peptides, the total energy is −124.2770 kcal/mol, with significant contributions from non-1,4 van der Waals interactions and dipole-dipole interactions. The 5-peptides have a total energy of −231.1312 kcal/mol, with non-1,4 van der Waals interactions and dipole-dipole interactions being the main stabilizing factors. The 15-peptides exhibit the lowest total energy of −792.9097 kcal/mol, indicating a highly stable structure. The energy decomposition analysis for the 15-peptides reveals the dominant role of non-1,4 van der Waals interactions, followed by dipole-dipole interactions and torsional energy in stabilizing the structure. Across all peptide sequences, the 1,4 van der Waals interactions and bending energy consistently oppose the stability. The interplay of these energy components provides insights into the structural and energetic properties of the studied peptide sequences.

**Energy Component**	**Value (kcal/mol)**
Stretch	1.2400
Bend	6.5174
Stretch-Bend	0.2652
Torsion	−9.0748
Non-1,4 VDW	−25.5240
1,4 VDW	14.5482
Dipole/Dipole	−9.7099
Total Energy	−21.7378
**3-peptides**	
Stretch	3.4936
Bend	22.4569
Stretch-Bend	0.7559
Torsion	−22.7554
Non-1,4 VDW	−113.0769
1,4 VDW	46.1615
Dipole/Dipole	−61.3128
Total Energy	−124.2770
**5-peptides**	
Stretch	5.5790
Bend:	39.5556
Stretch-Bend	1.3318
Torsion	-39.1375
Non-1,4 VDW	-197.7552
1,4 VDW	78.1562
Dipole/Dipole	-118.8611
Total Energy	-231.1312
**15-peptides**	
Stretch	16.1942
Bend:	123.6715
Stretch-Bend	3.9588
Torsion	-116.9006
Non-1,4 VDW	-637.9142
1,4 VDW	236.3119
Dipole/Dipole	-418.2313
Total Energy	-792.9097
